# Supplementary material for: Recognition Site Modifiable Macrocycle: Synthesis, Functional Group Variation and Structural Inspection
Source: Molecules. 2023 Jan 31;28(3):1338. doi: 10.3390/molecules28031338 (PMC9921963; doi:10.3390/molecules28031338)

# Supplementary Information

## Recognition site modifiable macrocycle: synthesis, functional group variation and structural inspection

Linmeng Fan, Min Du, Lichun Kong, Yan Cai and Xiaobo Hu*

Key Laboratory of the Ministry of Education for Advanced Catalysis Materials, College of Chemistry and Life Sciences, Zhejiang Normal University, 688 Yingbin Road, Jinhua 321004, P. R. China

* E-mail: xiaobo.hu@zjnu.edu.cn

**Table of contents**

**Materials, methods, and abbreviations** ---------------------------------- S2

**Synthesis and characterization** ------------------------------------------- S4

**2D NMR spectra** ---------------------------------------------------------- S12

**1D NMR spectra** ---------------------------------------------------------- S18

**Mass spectra** ---------------------------------------------------------------- S26

## Materials, methods, and abbreviations

Materials

All commercially available starting materials and reagents were used without further purification. Anhydrous THF and DCM were obtained from commercial sources. Analytical thin layer chromatography (TLC) was performed on silica gel plates (Merck 60F254) visualized with a UV lamp (254 nm). Column chromatography was performed with commercial glass columns using silica gel 200 - 300 mesh (particle size 0.045 - 0.075 mm).

Mass spectrometry

High resolution electrospray ionization time-of-flight (HRESI-TOF) mass spectra were measured in the positive ion mode on an Agilent 6230 mass spectrometer.

NMR spectroscopy

$^1$H NMR spectra were recorded on a Bruker Avance III HD 400 in $CDCl_3$ or DMSO-$d_6$. Chemical shifts are reported in ppm relative to the residual solvent signal of $CDCl_3$ ($\delta$ = 7.26 ppm) or DMSO-$d_6$ ($\delta$ = 2.50 ppm). Abbreviations used for signal multiplicity are: s = singlet, d = doublet, t = triplet, q = quartet, m = multiplet or overlap of nonequivalent resonances, br = broad. Coupling constants, *J*, are reported in Hertz (Hz). $^{13}C\{^1H\}$ NMR spectra were recorded on a Bruker AVANCE III HD 400 in $CDCl_3$ or DMSO-$d_6$ and the observed signals are reported in ppm relative to the residual solvent signal of $CDCl_3$ ($\delta$ = 77.16 ppm) or DMSO-$d_6$ ($\delta$ = 39.52 ppm).

2D NMR parameters.

Spectra of nuclear overhauser effect spectroscopy (NOESY) and correlation spectroscopy (COSY) experiments were recorded on a Bruker Avance III HD 400 by

means of a BBO (BB-H/F-D) probe. Data processing was performed with Topspin software. $^1$H-$^1$H NOESY acquisition of **10** was performed with a time domain size of 2048 (F2) × 256 (F1), 16 scans per increment, spectral width of 11.79 (F2) × 11.79 (F1) ppm, offset of 5.61 (F2) × 5.61 (F1), a pulse program of noesygpphpp, dwell time of 106 μs, relaxation delay of 2.04 s and a mixing time of 300 ms. $^1$H-$^1$H COSY acquisition of **10** was performed with a time domain size of 2048 (F2) × 128 (F1), 4 scans per increment, spectral width of 11.79 (F2) × 11.79 (F1) ppm, offset of 5.61 (F2) × 5.61 (F1), dwell time of 106 μs, relaxation delay of 1.98 s and a pulse program of cosygpmfqf.

Abbreviations

PyBop = (1H-Benzotriazol-1-yloxy) tripyrrolidinophosphonium hexafluorophosphate; DCM = dichloromethane; DMSO = dimethyl sulfoxide; THF = tetrahydrofuran; DMF = N,N-dimethylformamide; DIPEA = N,N-diisopropylethylamine; TFA = trifluoroacetic acid.

## Synthesis and characterization

Compounds **1**, **4** and **6** are commercially available and were used without further purification.

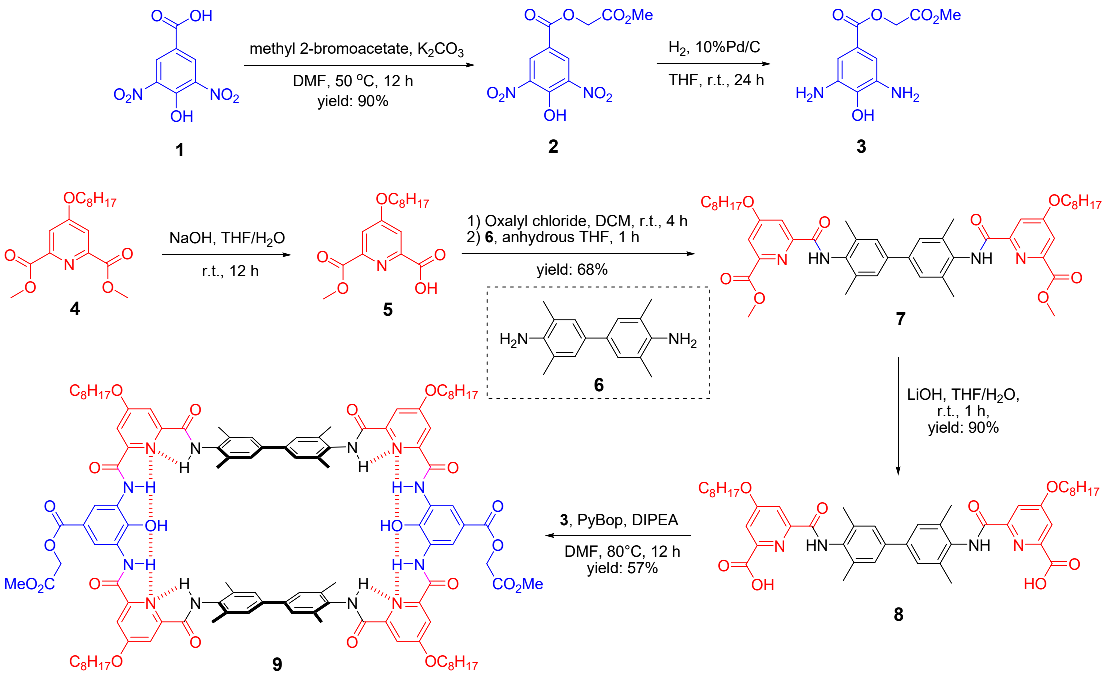


**Scheme S1**. Synthesis of macrocycle **9**.

Preparation of 2-methoxy-2-oxoethyl 4-hydroxy-3,5-dinitrobenzoate (**2**).

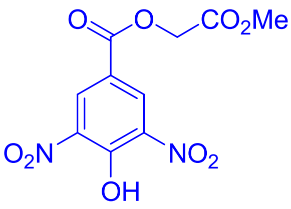


4-hydroxy-3, 5-dinitrobenzoic acid (compound **1**, 11.4 g, 50 mmol) and $K_2CO_3$ (10.36 g, 75 mmol) were suspended in 200 mL DMF, followed by the addition of methyl 2-bromoacetate (7.1 mL, 75 mmol) at 50 °C. The mixture was stirred at this temperature for 12 hours and cooled to room temperature. The reaction mixture was concentrated under vacuum and the residue was treated with water to allow the precipitation of the crude. The solid was filtered, dried and washed with methanol to afford the titled compound as a yellow solid (13.5 g, 90%). $^1$H NMR (400 MHz, $CDCl_3$) $\delta$ (ppm) = 11.82 (br, 1H), 9.00 (s, 2H), 4.93 (s, 2H), 3.82 (s, 3H). $^{13}$C{$^1$H} NMR (101 MHz, $CDCl_3$): $\delta$ (ppm) = 167.4, 162.0, 152.5, 137.5, 132.4, 120.7, 61.8, 52.6. HRMS (ESI): m/z calcd for $C_{10}H_8N_2O_9Na$ [M + Na]$^+$: 323.0122, found: 323.0118.

Preparation of 2-methoxy-2-oxoethyl 3,5-diamino-4-hydroxybenzoate (**3**).

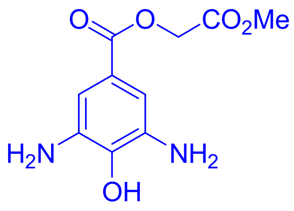


Compound **2** (5.1 g, 17 mmol) and 10% Pd/C (0.51 g) were suspended in 100 mL anhydrous THF under $N_2$. Then $N_2$ was exchanged with $H_2$ and the mixture was allowed to stir at room temperature for 24 hours. Afterwards, Pd/C was filtered and the solution was concentrated under vacuum to afford the titled compound as a dark green sticky solid (4.1 g, quantitative). Note: compound **3** is ready to be oxidized and thus it was immediately used in the next step without further purification.

Preparation of the diester (**7**)

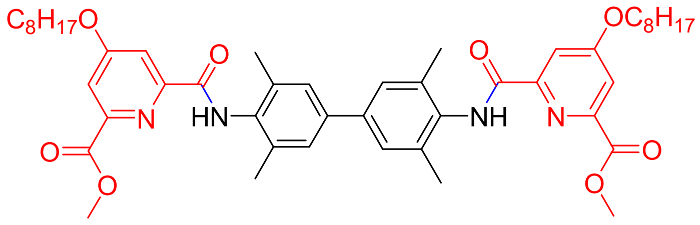


dimethyl 4-(octyloxy)pyridine-2,6-dicarboxylate (compound **4**, 8.1 g, 25 mmol) was dissolved in 50 mL THF followed by the addition of 5 M NaOH aqueous solution (5 mL, 25 mmol). The mixture was stirred at room temperature for 12 hours. THF was removed under vacuum and the reaction mixture was acidified by 1 M HCl. The resulting precipitation was collected and the solid was dried to give the crude of the intermediate **5**. Then, the intermediate **5** was dissolved in 100 mL anhydrous DCM followed by the addition of oxalyl chloride (4.23 mL, 50 mmol) under $N_2$. The solution was stirred at room temperature for four hours. The solvent and extra oxalyl chloride were removed under vacuum and re-dissolved in 50 mL anhydrous THF. Compound **6** (2.4 g, 10 mmol) and DIPEA (8.3 ml, 50 mmol) were dissolved in another 50 mL anhydrous THF. The two THF solutions were then mixed immediately and the mixture was stirred at room temperature under $N_2$ for 1 hour. Solvent was removed under vacuum and the crude product was washed with methanol and ethyl acetate to give the titled compound as a white solid (5.6 g, 68%). $^1$H NMR (400 MHz,

$CDCl_3$) $\delta$ (ppm) = 9.66 (s, 2H), 7.97 (d, $J$ = 2.0 Hz, 2H), 7.78 (d, $J$ = 2.0 Hz, 2H), 7.35 (s, 4H), 4.17 (t, $J$ = 6.4 Hz, 4H), 4.01 (s, 6H), 2.36 (s, 12H), 1.85 (m, 4H), 1.48 (m, 4H), 1.30 (m, 16H), 0.89 (t, $J$ = 6.4 Hz, 6H). $^{13}C\{^1H\}$ NMR (101 MHz, $CDCl_3$): $\delta$ (ppm) = 167.7, 165.1, 161.9, 151.9, 148.1, 139.8, 135.6, 132.9, 127.0, 115.0, 110.9, 69.1, 52.9, 31.8, 29.22, 29.19, 28.8, 25.9, 22.7, 18.8, 14.1. HRMS (ESI): m/z calcd for $C_{48}H_{63}N_4O_8$ $[M + H]^+$: 823.4640 found: 823.4643.

Preparation of the diacid (**8**)

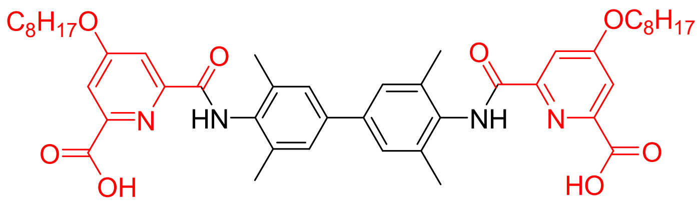


Compound **7** (4.1 g, 5 mmol) was dissolved in 20 mL THF and followed by the addition of 5 M LiOH aqueous solution (3 mL, 15 mmol). The mixture was stirred at room temperature for one hour and THF was removed under vacuum. The residue was acidified by 1 M HCl and the resulting solid was filtered and dried to give the titled compound as a white solid (3.6 g, 90%). $^1H$ NMR (400 MHz, DMSO-$d_6$) $\delta$ (ppm) = 12.98 (br, 2H), 10.72 (s, 2H), 7.82 (d, $J$ = 2.4 Hz, 2H), 7.76 (d, $J$ = 2.4 Hz, 2H), 7.53 (s, 4H), 4.27 (t, $J$ = 6.4 Hz, 4H), 2.28 (s, 12H), 1.78 (m, 4H), 1.44 (m, 4H), 1.27 (m, 19H, overlapped with grease signal), 0.86 (t, $J$ = 6.8 Hz, 6H). $^{13}C\{^1H\}$ NMR (101 MHz, 10% $CD_3OD/CDCl_3$): $\delta$ (ppm) = 167.9, 166.5, 162.7, 151.6, 148.3, 139.7, 135.9, 133.0, 126.8, 114.4, 111.8, 69.2, 31.7, 29.2, 28.7, 25.8, 22.6, 18.4, 14.0. HRMS (ESI): m/z calcd for $C_{46}H_{59}N_4O_8$ $[M + H]^+$: 795.4327 found: 795.4323.

Synthesis of the macrocycle (**9**)

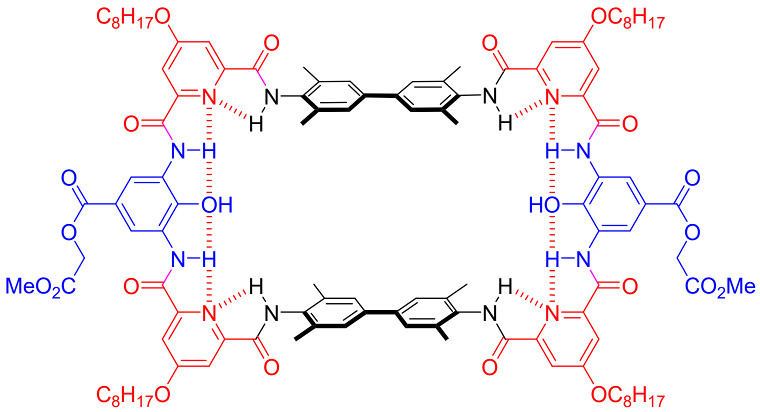


Compound **3** (0.6 g, 2.5 mmol), compound **8** (2.0 g, 2.5 mmol), PyBop (3.9 g, 7.5 mmol), and DIPEA (1.7 mL, 10 mmol) were dissolved in 200 mL DMF. The mixture was stirred at 80 °C for 12 hours. The reaction mixture was concentrated under vacuum and followed by the addition of 1M HCl to adjust the pH to 1-2. The resulting solid was collected by filtration and purified by column chromatography (eluent: DCM/MeOH = 10:1 v/v). The crude product was further washed with methanol to afford the titled compound as a yellow solid (1.43 g, 57%). $^1$H NMR (400 MHz, DMSO-$d_6$) $\delta$ (ppm) = 10.88 (s, 4H), 10.20 (s, 4H), 8.79 (s, 4H), 7.85 (s, 4H), 7.74 (s, 4H), 7.38 (s, 8H), 4.81 (s, 4H), 4.28 (s, 8H), 3.71 (s, 6H), 2.26 (s, 24H), 1.79 (s, 8H), 1.46 (m, 58H, overlapped with grease signal), 0.87 (m, 14H, overlapped with grease signal). $^{13}$C{$^1$H} NMR (101 MHz, 10% DMSO-$d_6$/$CDCl_3$): $\delta$ (ppm) = 169.4, 167.8, 167.1, 162.6, 161.2, 151.7, 151.2, 139.3, 136.2, 133.8, 127.2, 126.6, 118.0, 116.1, 111.3, 111.2, 68.9, 60.6, 52.1, 40.2, 39.8, 39.4, 36.4, 31.6, 29.5, 29.1, 28.7, 25.7, 22.5, 18.5, 14.0. HRMS (ESI): m/z calcd for $C_{112}H_{134}N_{12}O_{22}$ [M + 2H]$^{2+}$: 999.9885 found: 999.9885.

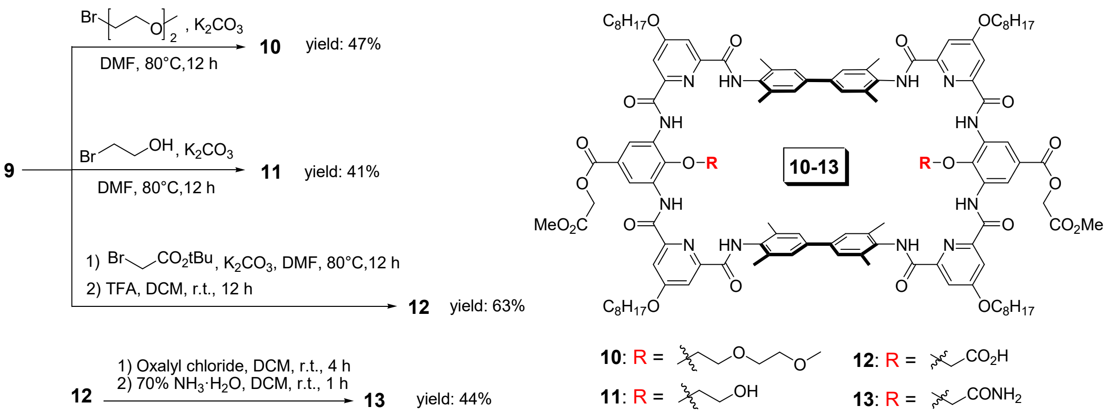


**Scheme S2**. Synthesis of macrocycles **10-13**.

Synthesis of the macrocycle (**10**)

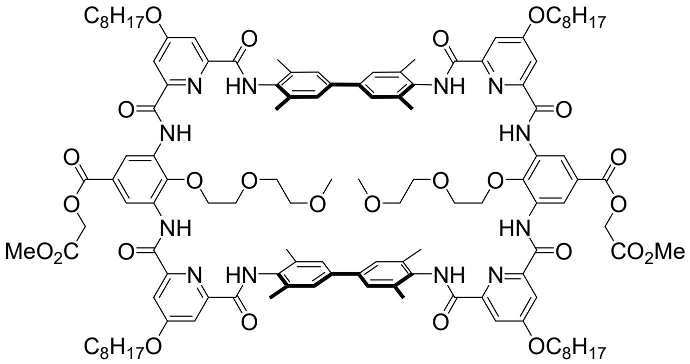


Compound **9** (100 mg, 0.05 mmol), $K_2CO_3$ (21 mg, 0.15 mmol) were suspended in 5 mL DMF, followed by the addition of 1-Bromo-2-(2-methoxyethoxy) ethane (0.34 mL, 0.25 mmol). The mixture was stirred at 80 °C for 12 hours. Then 1 M HCl aqueous solution was added and the resulting solid was collected by filtration. The crude product was further purified by column chromatography (eluent: DCM/MeOH = 50:1 v/v) to afford the titled compound as a pale pink solid (52 mg, 47%). $^1$H NMR (400 MHz, $CDCl_3$) $\delta$ (ppm) = 10.34 (s, 4H), 9.35 (s, 4H), 9.17 (s, 4H), 8.04 (d, $J$ = 2.4 Hz, 4H), 7.99 (d, $J$ = 2.4 Hz, 4H), 7.39 (s, 8H), 4.92 (s, 4H), 4.36 (br, 4H), 4.21 (t, $J$ = 6.4 Hz, 8H), 3.82 (s, 6H), 3.60 (br, 4H), 3.38 – 3.26 (m, 4H), 3.15 – 3.06 (m, 4H), 2.99 (s, 6H), 2.31 (s, 24H), 1.87 (m, 8H), 1.52 – 1.21 (m, 49H, overlapped with water signal), 0.89 (t, $J$ = 6.4 Hz, 12H). $^{13}C\{^1H\}$ NMR (101 MHz, $CDCl_3$): $\delta$ (ppm) = 168.4, 168.2, 165.2, 161.8, 161.4, 150.9, 150.6, 140.9, 140.0, 135.6, 132.9, 131.6, 127.2, 126.5, 118.6, 112.2,112.1, 71.6, 70.0, 69.4, 68.7, 61.3, 58.6, 52.3, 31.8, 29.4, 29.2, 29.16, 28.8, 25.8, 22.7, 18.6, 14.1. HRMS (ESI): m/z calcd for $C_{122}H_{154}N_{12}O_{26}$ $[M + 2H]^{2+}$: 1102.0566 found:1102.0539.

Synthesis of the macrocycle (**11**)

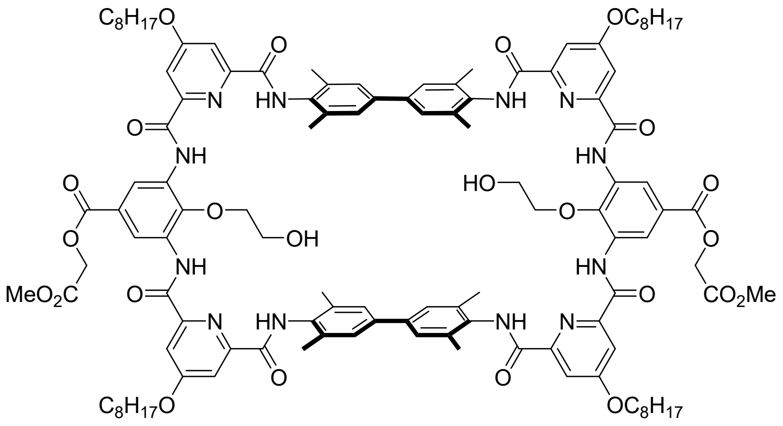


Compound **9** (100 mg, 0.05 mmol), $K_2CO_3$ (21 mg, 0.15 mmol) were suspended in 5 mL DMF, followed by the addition of 2-bromoethanol (0.18 mL, 0.25 mmol). The mixture was stirred at 80 °C for 12 hours. Then 1 M HCl aqueous solution was added and the resulting solid was collected by filtration. The crude product was further purified by column chromatography (eluent: DCM/MeOH = 50:1 v/v) to afford the titled compound as a white solid (43 mg, 41%). $^1$H NMR (400 MHz, DMSO-*d*$_6$) $\delta$ (ppm) = 11.19 (s, 4H), 10.76 (s, 4H), 8.85 (s, 4H), 7.89 (d, *J* = 2.4 Hz, 4H), 7.78 (m, 12H), 6.02 (t, *J* = 4.8 Hz, 2H), 5.01 (s, 4H), 4.28 (t, *J* = 6.0 Hz, 8H), 4.16 (br, 4H), 3.74 (s, 6H), 2.24 (s, 24H), 1.78 (m, 8H), 1.43 (m, 8H), 1.26 (m, 34H, overlapped with grease signal), 0.85 (t, *J* = 6.4 Hz, 13H, overlapped with grease signal). $^{13}$C{$^1$H} NMR (101 MHz, 2%DMSO-*d*$_6$/$CDCl_3$): $\delta$ (ppm) = 168.4, 168.1, 165.1, 162.4, 161.8, 151.3, 150.4, 142.7, 139.7, 136.1, 133.5, 131.4, 126.9, 125.7, 118.9, 112.1, 111.8, 69.3, 61.3, 60.6, 52.2, 31.8, 29.2, 29.2, 28.8, 25.8, 22.6, 18.6, 14.1. HRMS (ESI): m/z calcd for $C_{116}H_{142}N_{12}O_{24}$ [M + 2H]$^{2+}$: 1044.0147 found: 1044.0139.

Synthesis of the macrocycle (**12**)

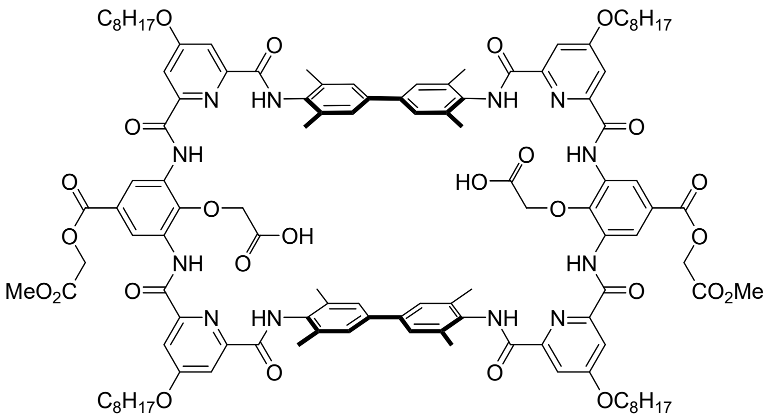


Compound **9** (200 mg, 0.1 mmol), $K_2CO_3$ (42 mg, 0.3 mmol) were suspended in 10 mL DMF, followed by the addition of tert-butyl bromoacetate (0.73 mL, 0.50 mmol). The mixture was stirred at 80 °C for 12 hours. Then 1 M HCl aqueous solution was added and the resulting solid was collected by filtration and was further purified by column chromatography (eluent: DCM/MeOH = 50:1 v/v). The intermediate was then treated with TFA (1 mL) in DCM (4 mL) for 12 hours. The reaction mixture was concentrated under vacuum and then washed by ether and methanol to afford the titled compound as a white solid (133 mg, 63%). $^{1}$H NMR (400 MHz, DMSO-$d_6$) $\delta$ (ppm) = 12.41 (s, 4H), 11.40 (s, 4H), 8.82 (s, 4H), 7.85 (s, 4H), 7.77 (s, 4H), 7.64 (s, 8H), 5.00 (s, 4H), 4.28 (br, 8H), 4.20 (br, 4H), 3.74 (s, 6H), 2.24 (s, 24H), 1.79 (br, 8H), 1.45 (br, 8H), 1.27 (m, 45H, overlapped with grease signal), 0.86 (m, 14H, overlapped with grease signal). $^{13}$C{$^{1}$H} NMR (101 MHz, 2%DMSO-$d_6$/$CDCl_3$): $\delta$ (ppm) = 168.3, 168.2, 165.1, 162.5, 162.0, 151.1, 150.7, 139.3, 139.1, 136.4, 133.6, 130.8, 126.6, 126.5, 112.0, 112.3, 112.29, 111.9, 69.2, 61.3, 52.3, 31.8, 29.7, 29.2, 29.19, 28.8, 25.8, 22.6, 18.6, 14.1. HRMS (ESI): m/z calcd for $C_{116}H_{138}N_{12}O_{26}$ [M + 2H]$^{2+}$: 1057.9940 found:1057.9937.

Synthesis of the macrocycle (**13**)

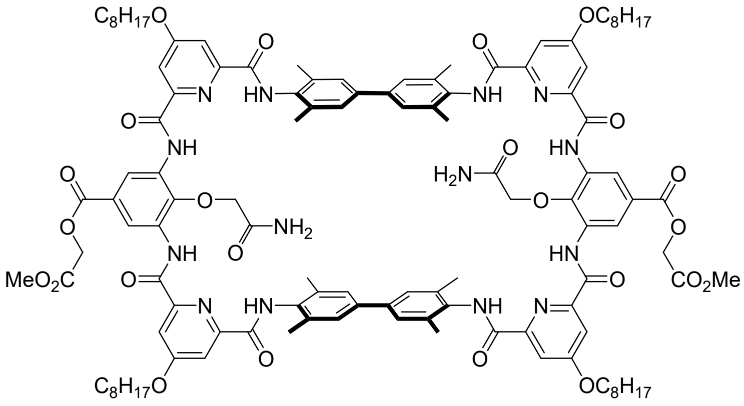


Compound **12** (73 mg, 0.034 mmol) was suspended in 5 mL DCM. Oxalyl chloride (0.15 mL, 0.173 mmol) was added at room temperature. The mixture was stirred at this temperature for 4 hours. DCM and the extra oxalyl chloride were removed under vacuum and re-dissolved with 5 mL DCM. Ammonium hydroxide (0.06 mL, 0.173 mmol) was then added and the mixture was stirred at room temperature for one hour. Solvents were removed under vacuum and the resulting solid was washed with methanol to give the titled compound as a pale yellow solid (32 mg, 44%). $^1$H NMR (400 MHz, DMSO-$d_6$) $\delta$ (ppm) = 11.56 (s, 4H), 10.79 (s, 4H), 8.88 (s, 4H), 7.91 (s, 4H), 7.81 (s, 4H), 7.73 (s, 8H), 5.01 (br, 4H), 4.63 (br, 4H), 4.29 (br, 8H), 3.74 (s, 6H), 2.24 (s, 24H), 1.79 (br, 8H), 1.44 (br, 8H), 1.27 (m, 42H, overlapped with grease signal), 0.86 (m, 17H, overlapped with grease signal). $^{13}$C{$^1$H} NMR (101 MHz, 5%DMSO-$d_6$/CDCl$_3$): $\delta$ (ppm) = 170.8, 168.3, 168.2, 165.3, 162.1, 151.2, 150.5, 139.6, 136.1, 133.4, 131.1, 126.6, 126.0, 119.4, 112.1, 112.0, 69.2, 61.3, 52.3, 31.7, 29.6, 29.2, 29.19, 29.14, 28.7, 25.8, 22.6, 18.4, 14.0. HRMS (ESI): m/z calcd for $C_{116}H_{139}N_{14}O_{24}Na$ [M + H + Na]$^{2+}$: 1068.0009 found:1067.9991.

## 2D NMR spectra

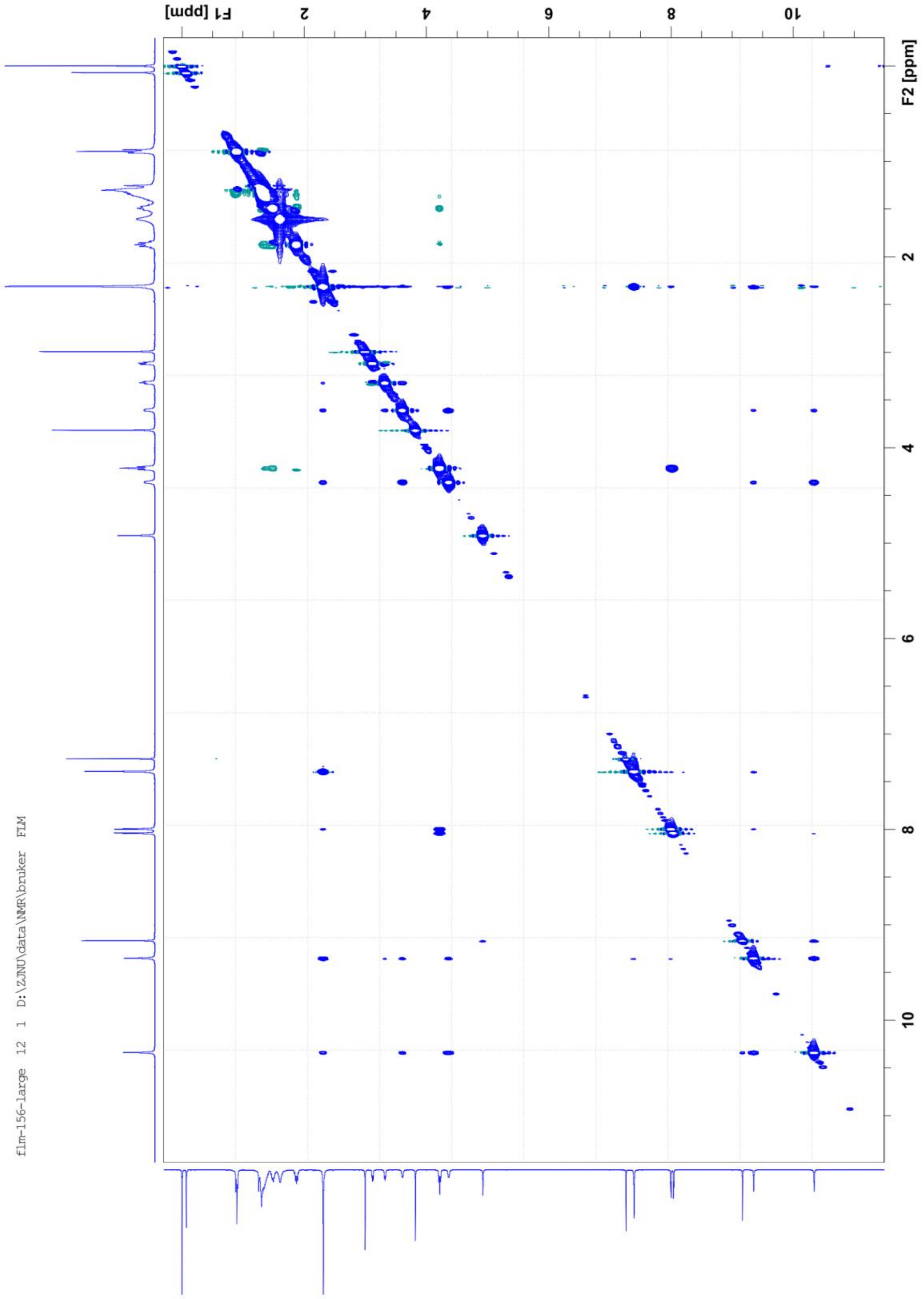


**Figure S1.** The $^1H$-$^1H$ NOESY (400 MHz) spectrum of **10** (5 mM) in $CDCl_3$ at 298 K.

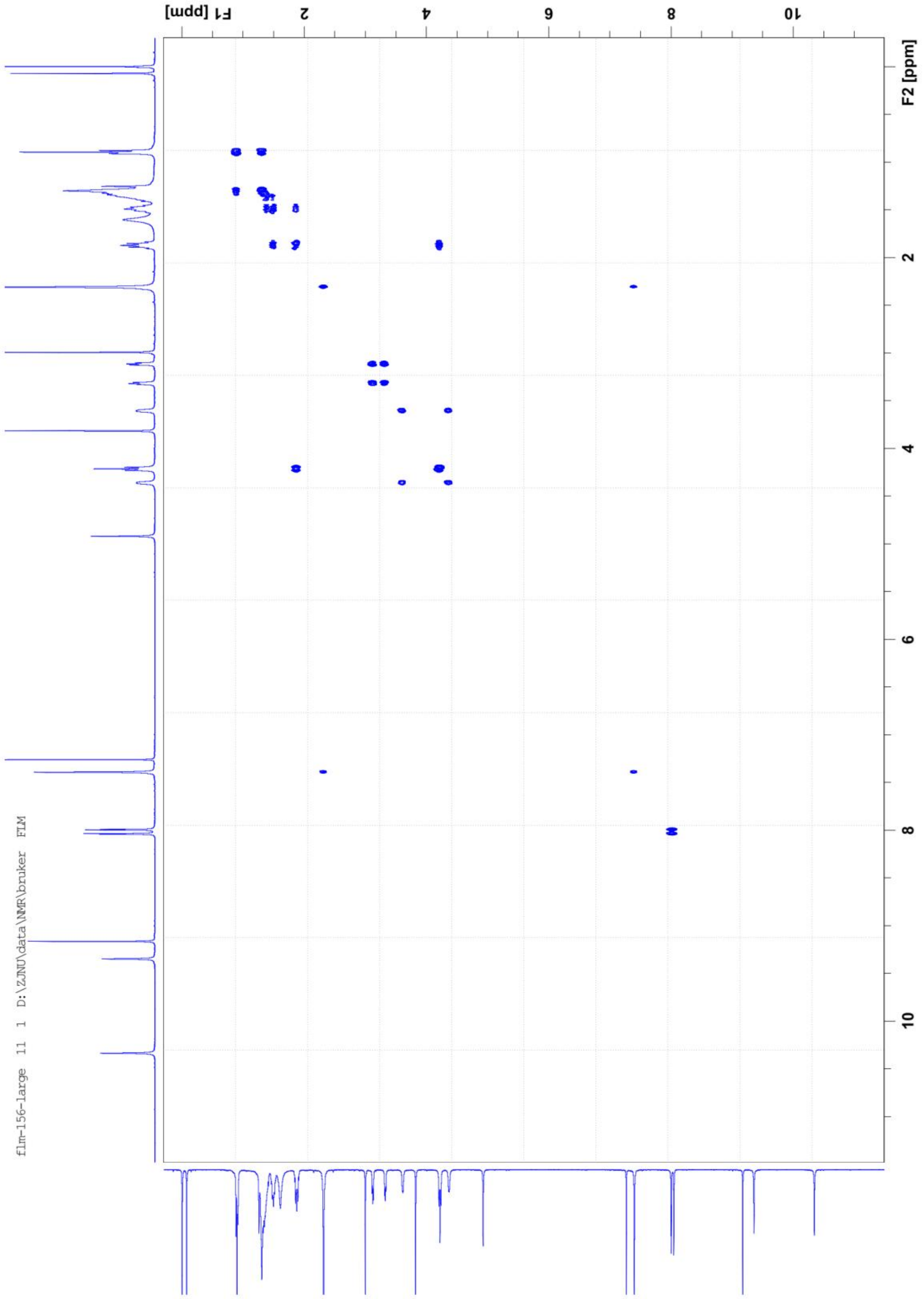


**Figure S2.** The $^{1}H$-$^{1}H$ COSY (400 MHz) spectrum of **10** (5 mM) in $CDCl_3$ at 298 K.

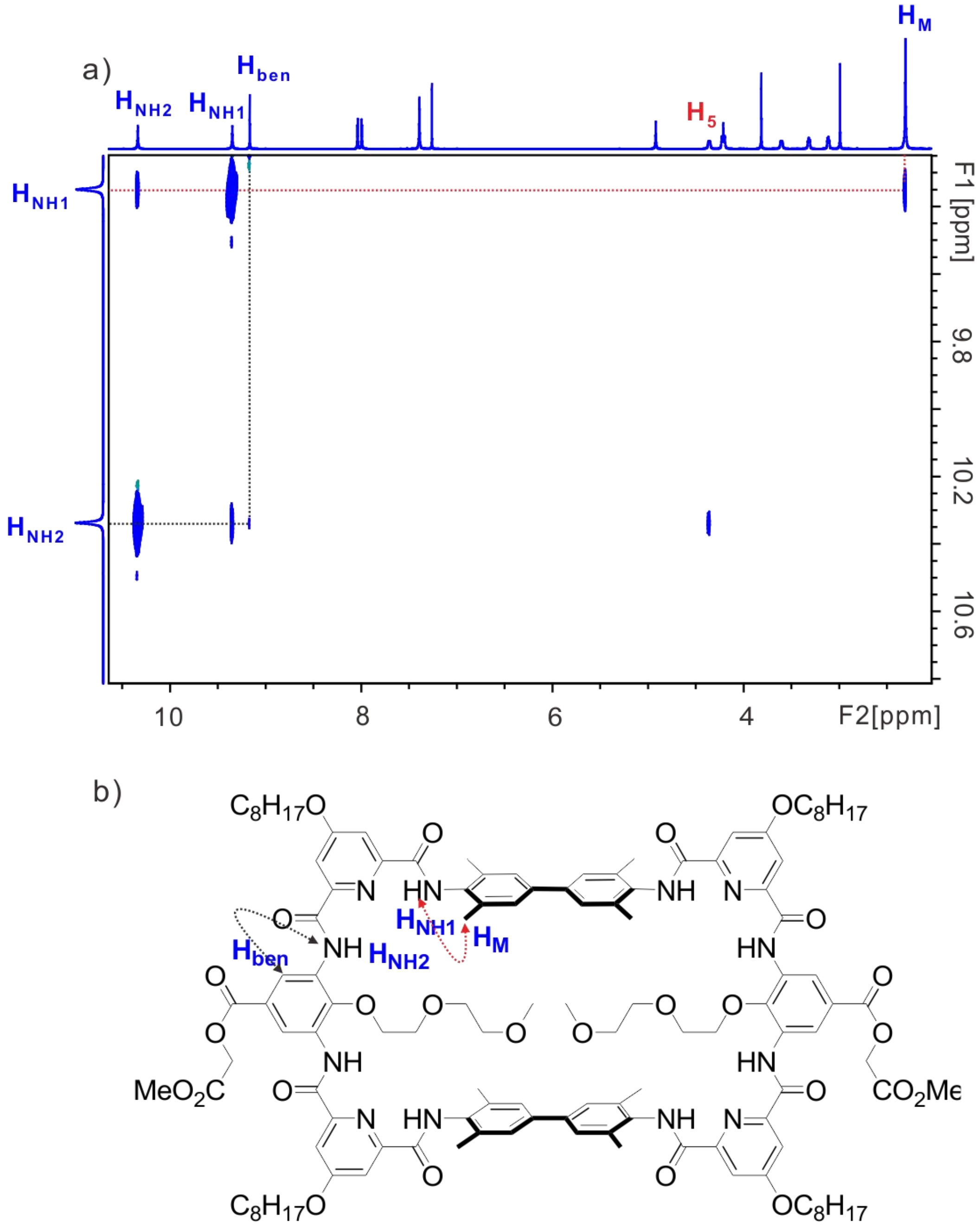


**Figure S3.** (a) the $^1H$-$^1H$ NOESY (400 MHz) spectrum of **10** (5 mM) in $CDCl_3$ at 298 K. (b) shows the corresponding correlations in the structure of **10**.

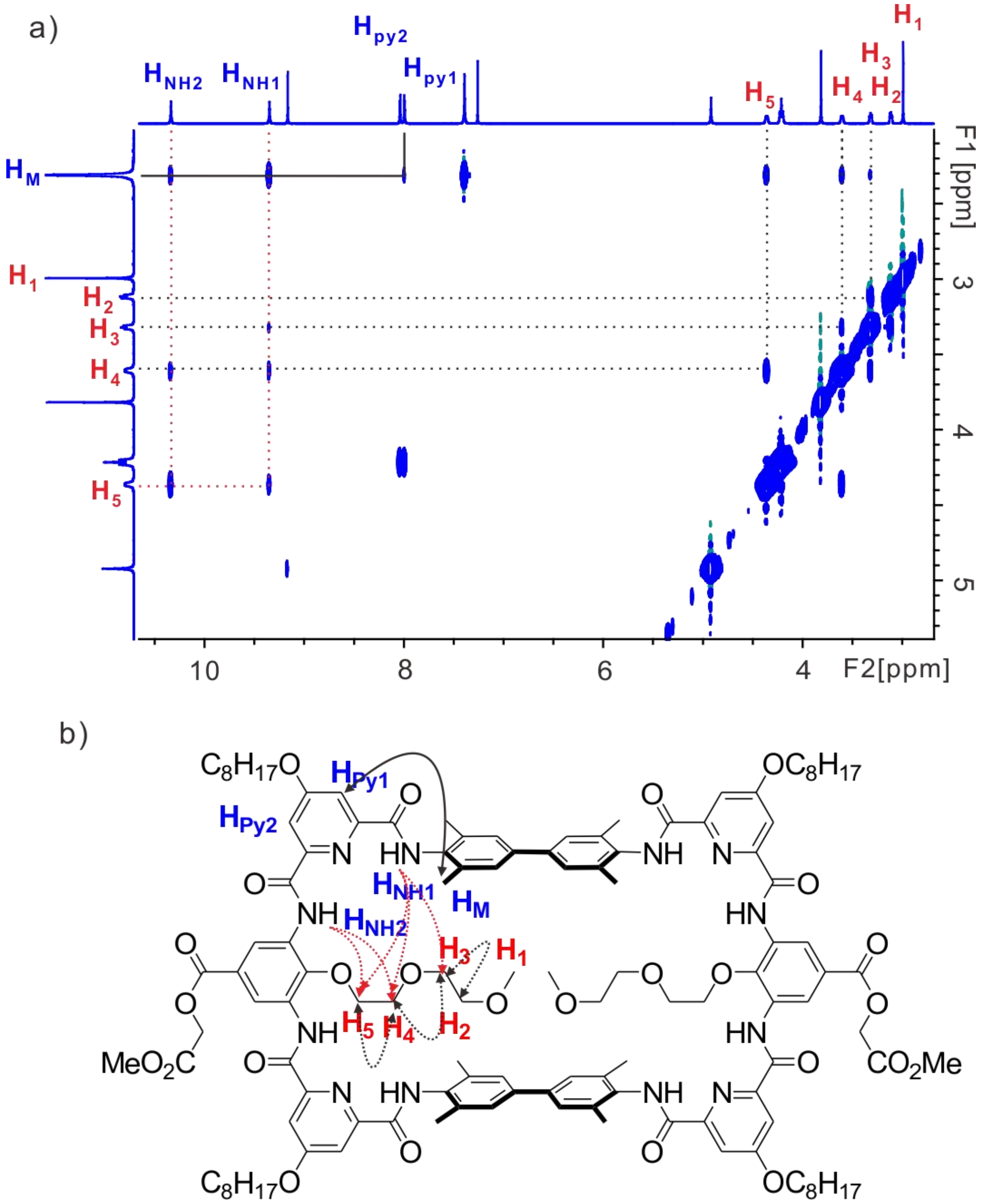


**Figure S4.** (a) the $^{1}$H-$^{1}$H NOESY (400 MHz) spectrum of **10** (5 mM) in $CDCl_3$ at 298 K. (b) shows the corresponding correlations in the structure of **10**.

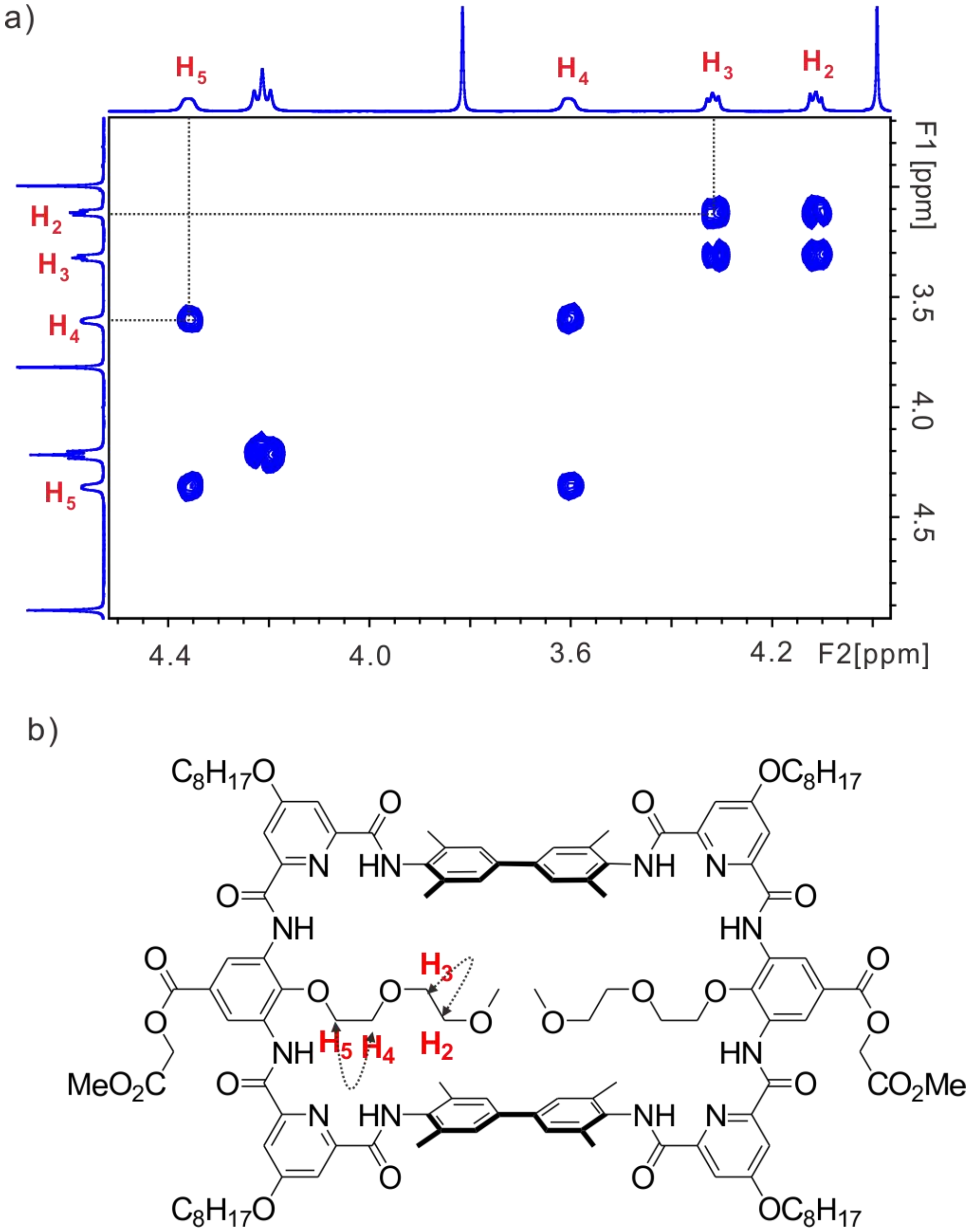


**Figure S5.** (a) the $^{1}$H-$^{1}$H COSY (400 MHz) spectrum of **10** (5 mM) in $CDCl_3$ at 298 K. (b) shows the corresponding correlations in the structure of **10**.

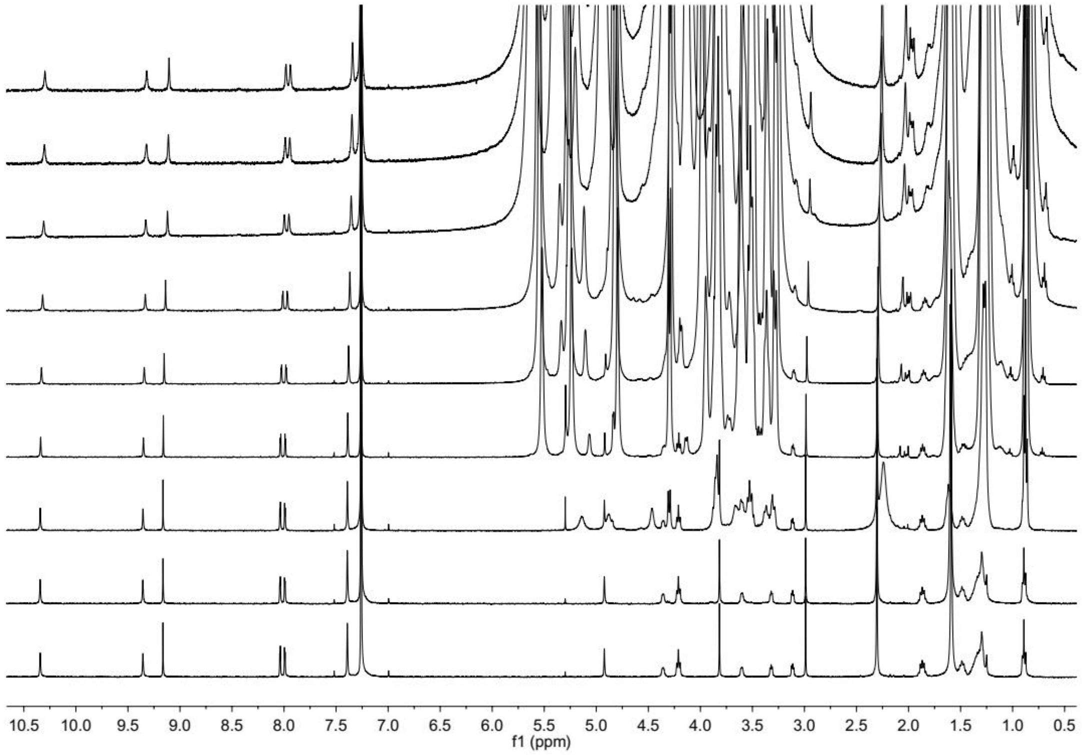


**Figure S6**. The full $^{1}H$ NMR spectra of the NMR titration study. From bottom to top, **10** in $CDCl_3$ (1 mM) titrated with n-octyl-β-D-glucopyranoside (**14**) from 1 to 500 equivalents.

## 1D NMR spectra

$^1H$ NMR (400 MHz, $CDCl_3$) spectrum of 2-methoxy-2-oxoethyl 4-hydroxy-3,5-dinitrobenzoate (**2**).

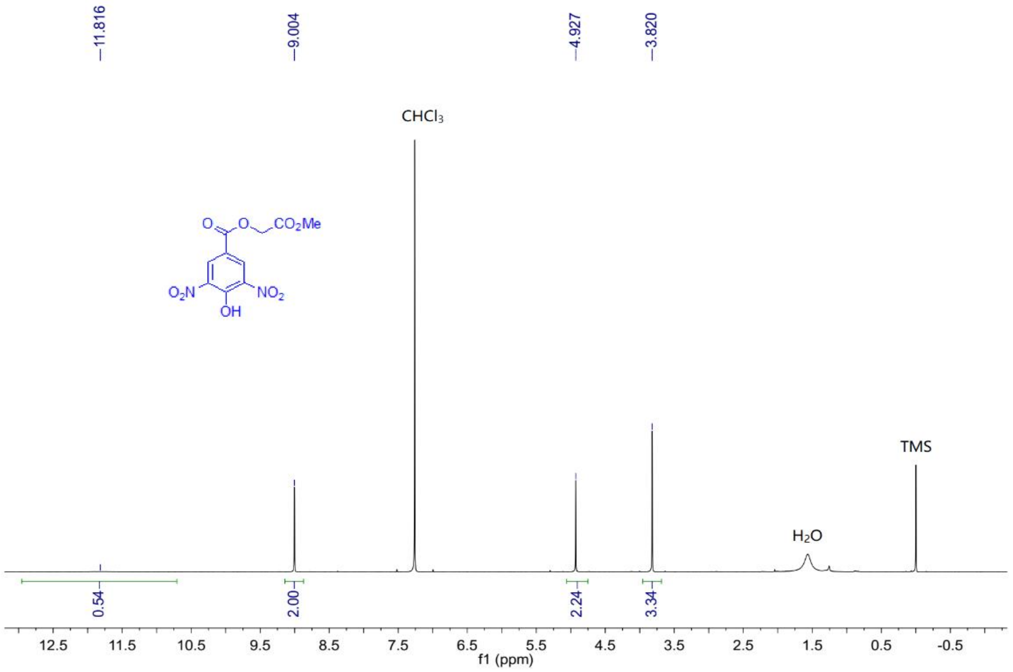


$^{13}C\{^1H\}$ NMR (101 MHz, $CDCl_3$) spectrum of 2-methoxy-2-oxoethyl 4-hydroxy-3,5-dinitrobenzoate (**2**).

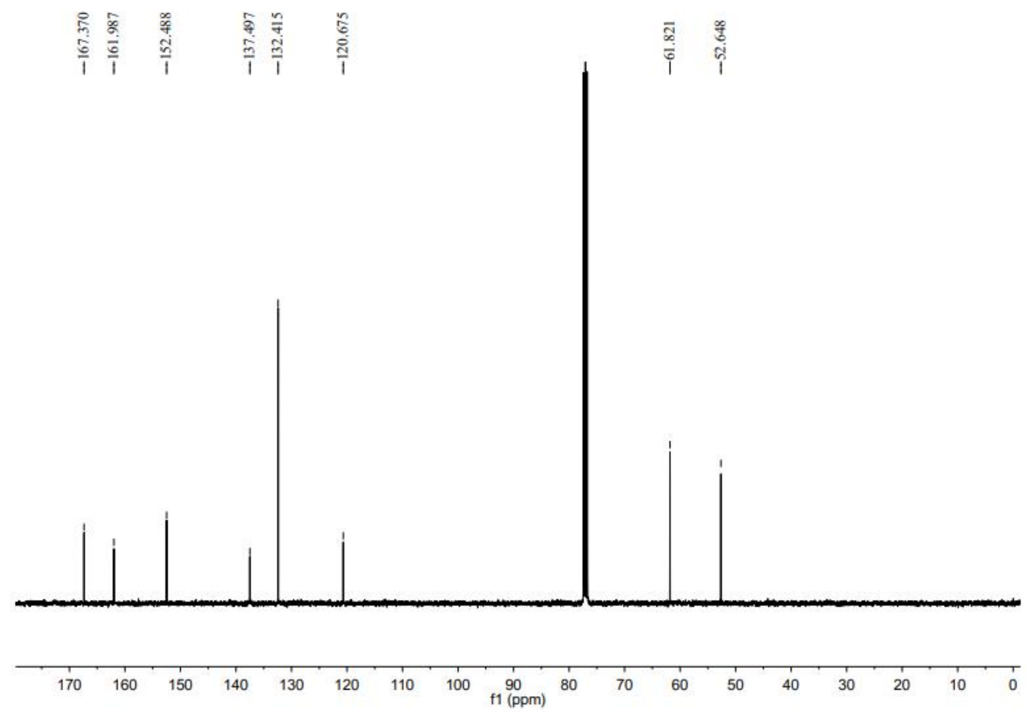

$^{1}H$ NMR (400 MHz, $CDCl_3$) spectrum of the diester (**7**).

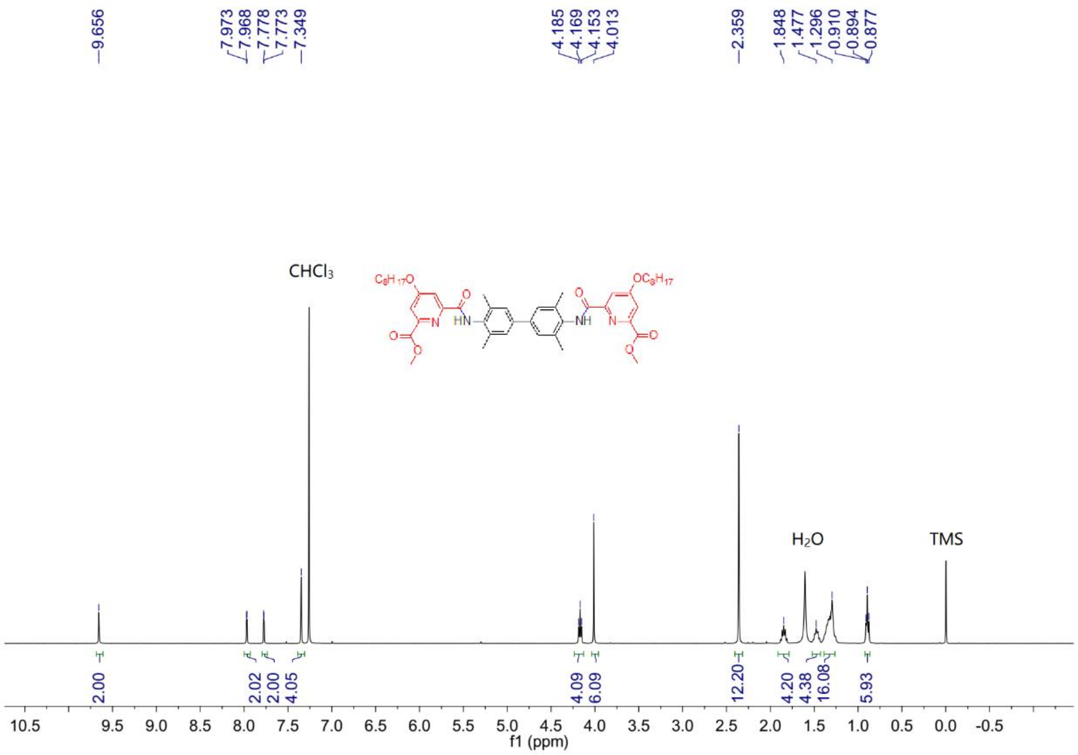


$^{13}C\{^{1}H\}$ NMR (101 MHz, $CDCl_3$) spectrum of the diester (**7**).

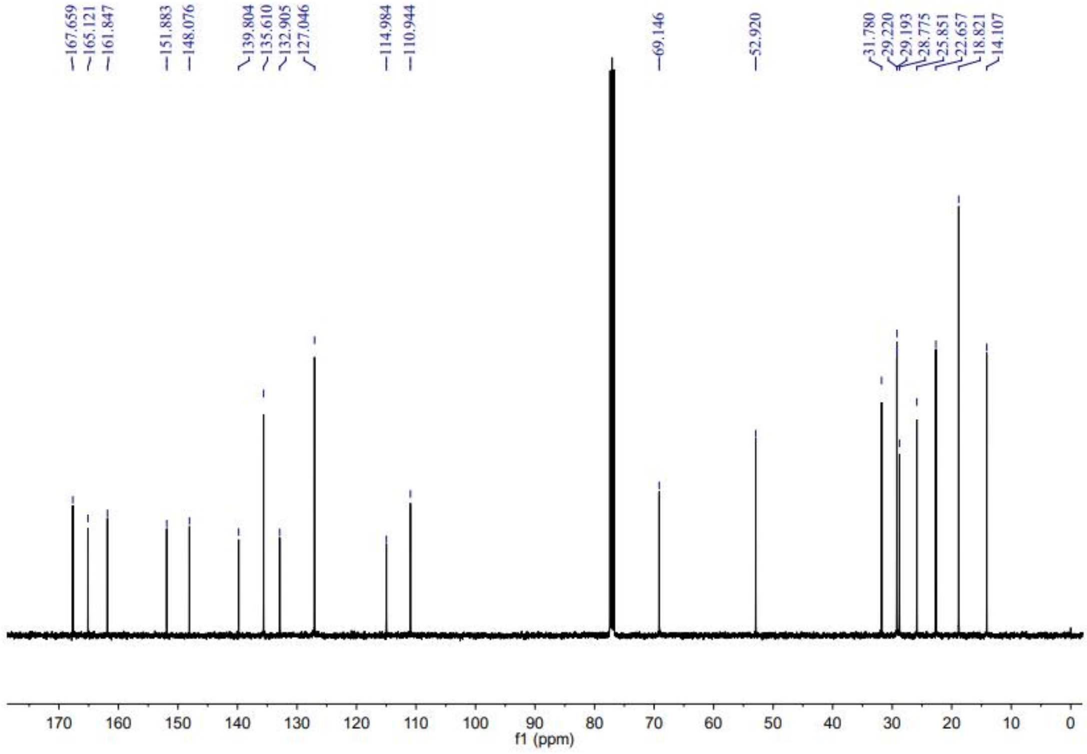

$^{1}H$ NMR (400 MHz, DMSO-$d_6$) spectrum of the diacid (**8**).

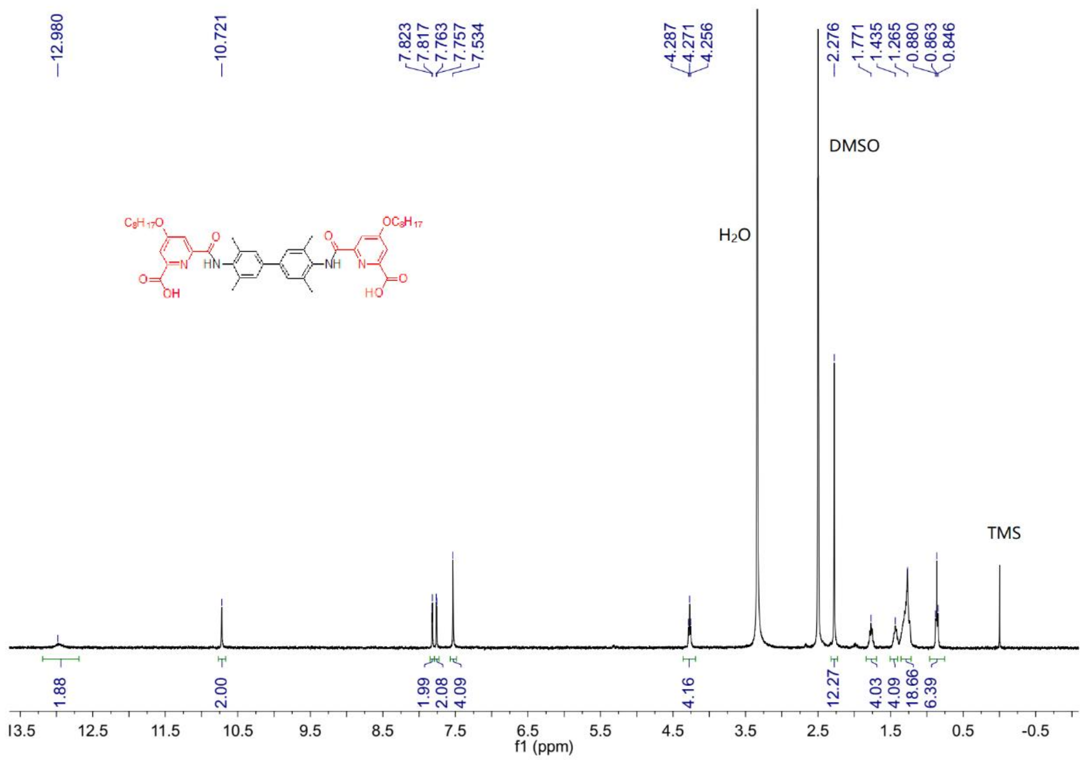


$^{13}C\{^{1}H\}$ NMR (101 MHz, 10% $CD_3OD/CDCl_3$) spectrum of the diacid (**8**).

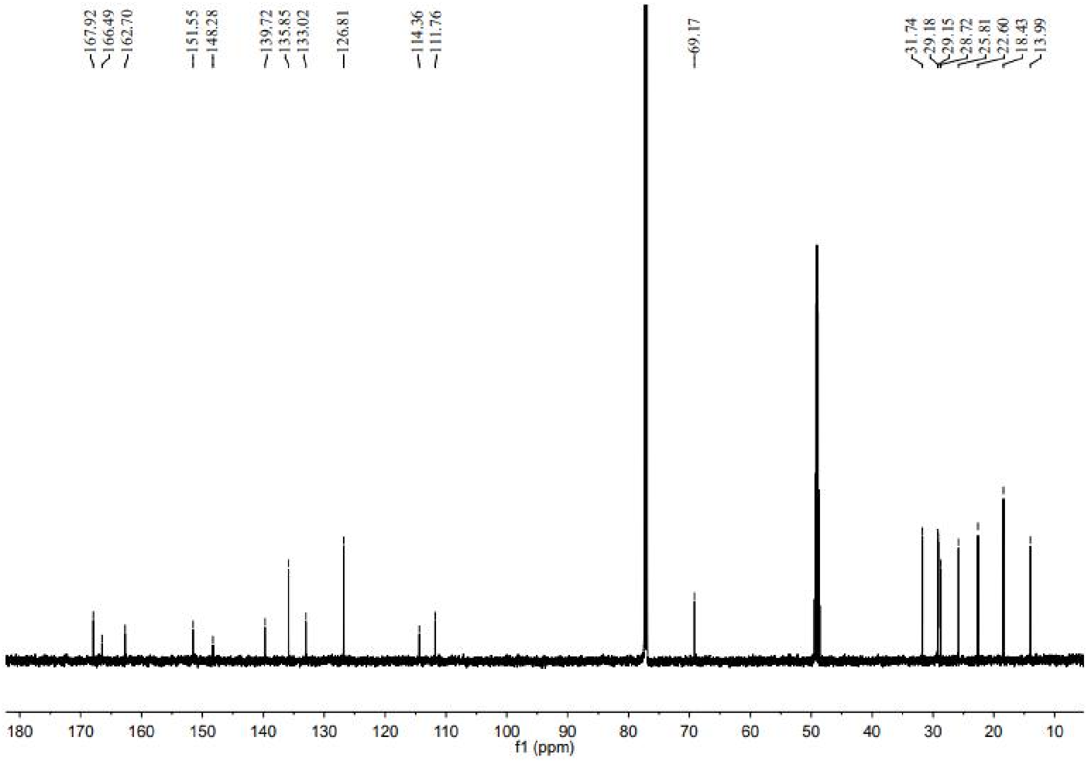

$^{1}H$ NMR (400 MHz, DMSO-$d_6$) spectrum of the macrocycle (**9**).

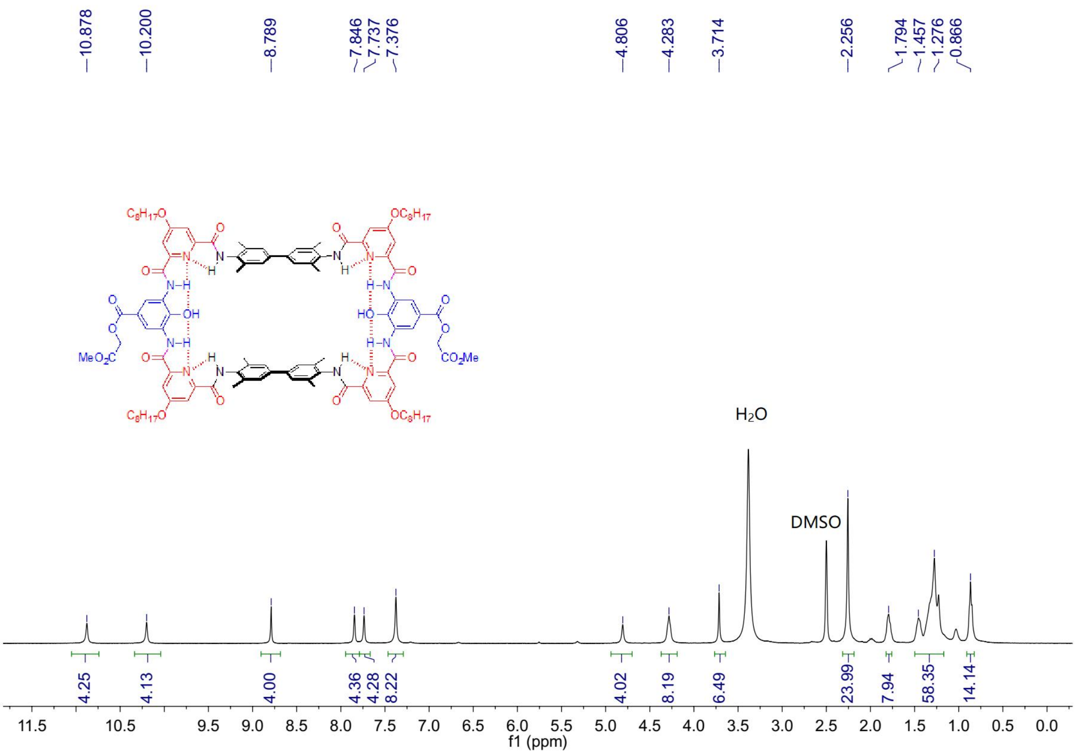


$^{13}C\{^{1}H\}$ NMR (101 MHz, 10% DMSO-$d_6$/$CDCl_3$) spectrum of the macrocycle (**9**).

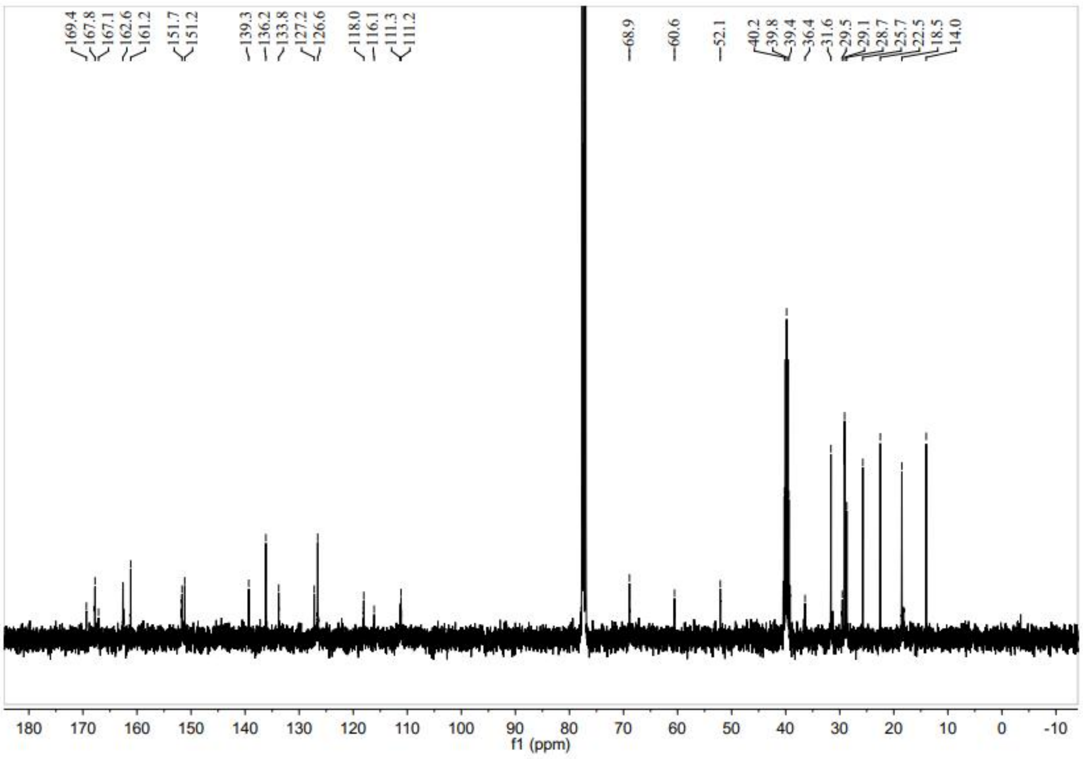

$^{1}H$ NMR (400 MHz, $CDCl_3$) spectrum of the macrocycle (**10**).

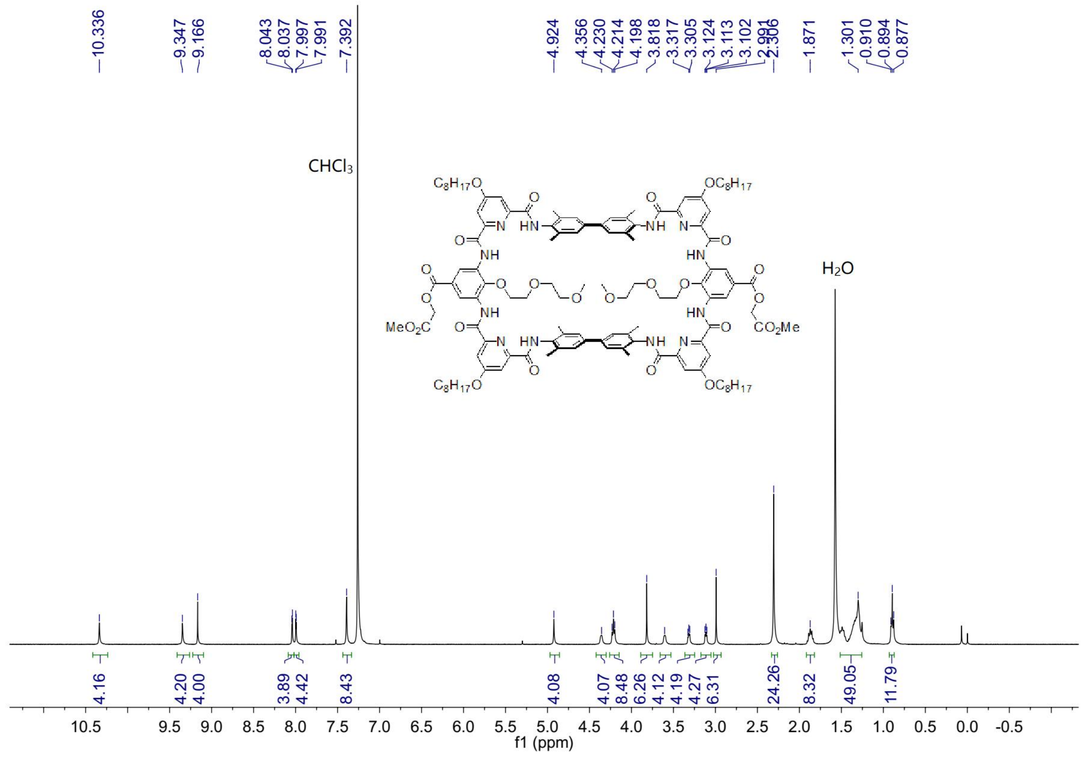


$^{13}C\{^{1}H\}$ NMR (101 MHz, $CDCl_3$) spectrum of the macrocycle (**10**).

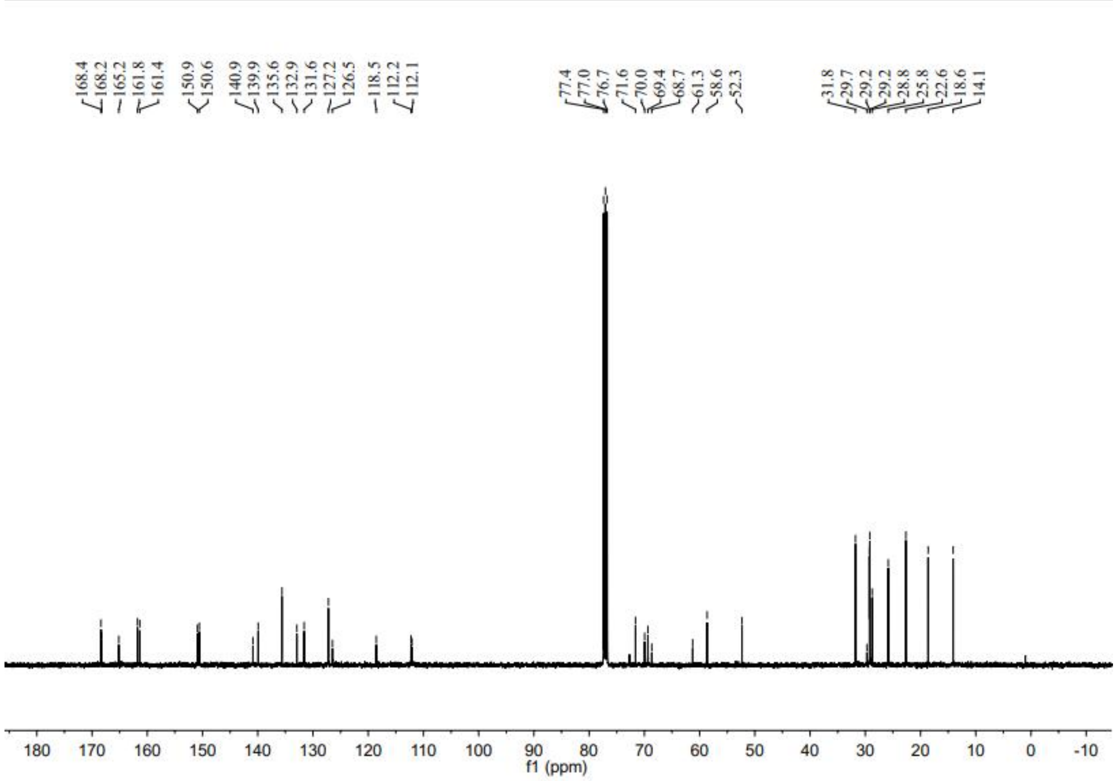

$^{1}H$ NMR (400 MHz, DMSO-$d_6$) spectrum of the macrocycle (**11**).

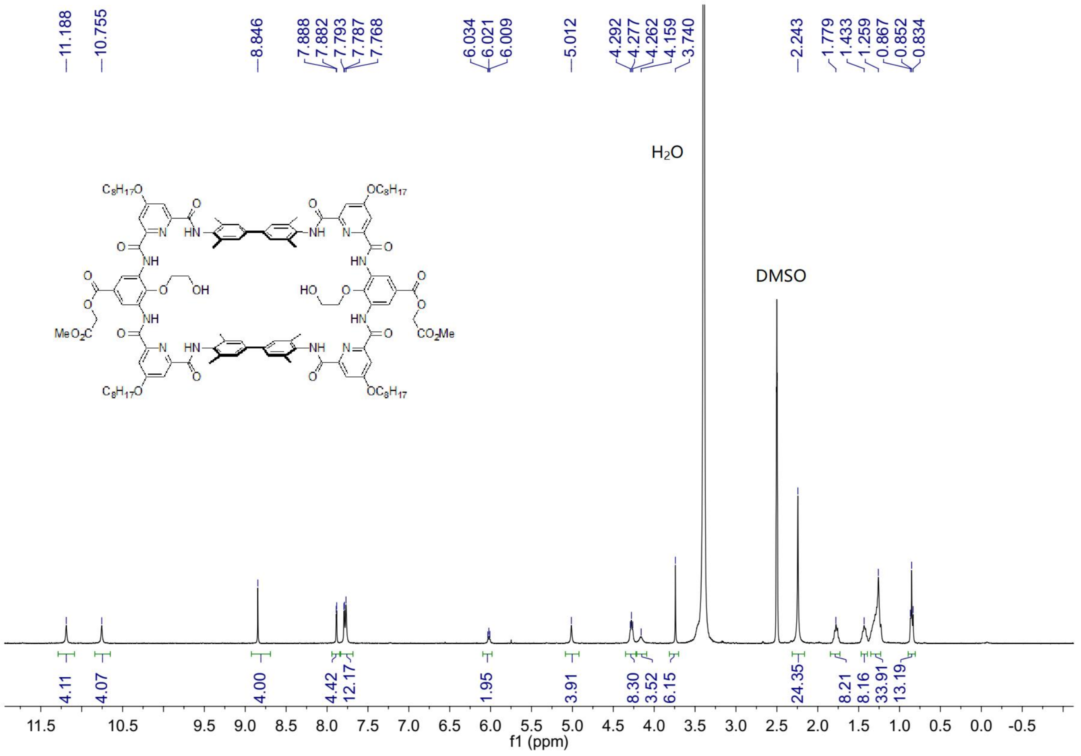

$^{13}C\{^{1}H\}$ NMR (101 MHz, 2%DMSO-$d_6$/$CDCl_3$) spectrum of the macrocycle (**11**).

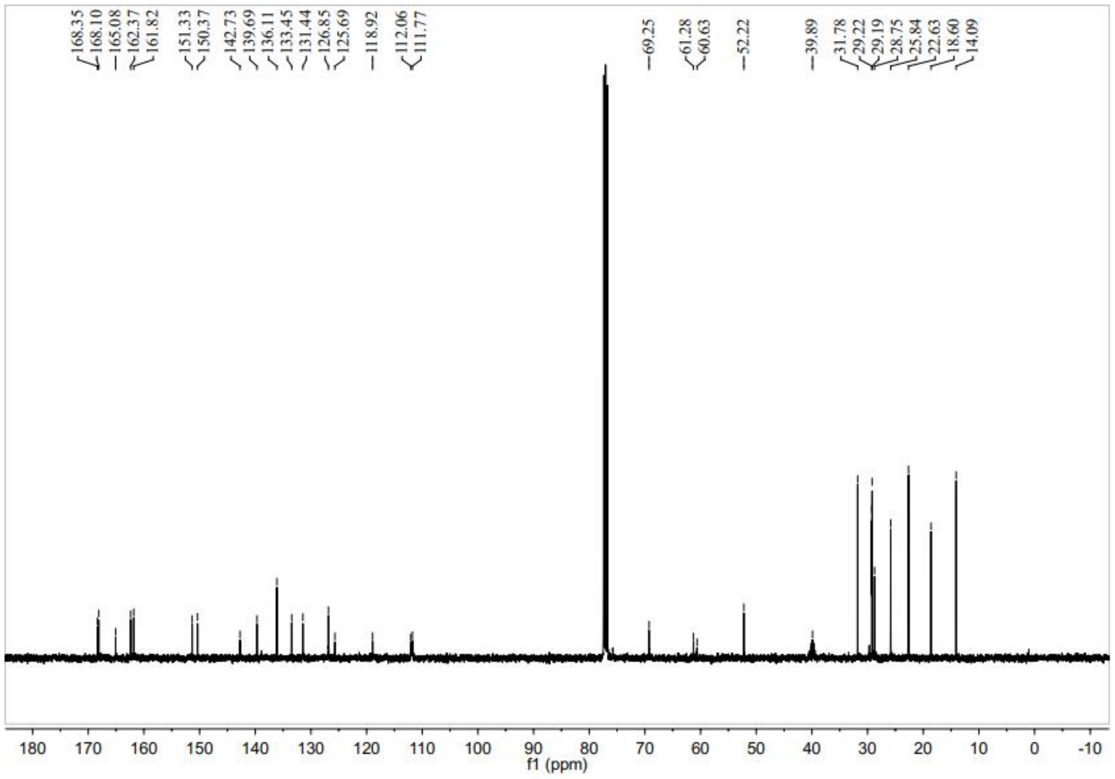

$^{1}H$ NMR (400 MHz, DMSO-$d_6$) spectrum of the macrocycle (**12**).

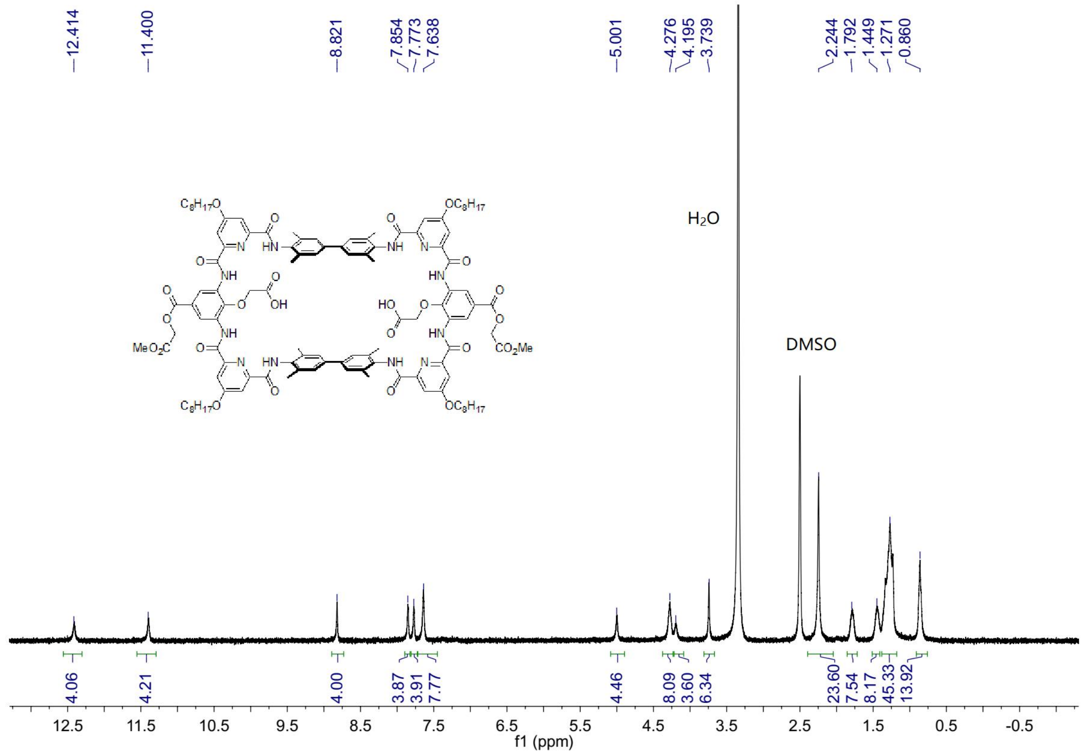


$^{13}C\{^{1}H\}$ NMR (101 MHz, 2%DMSO-$d_6$/$CDCl_3$) spectrum of the macrocycle (**12**).

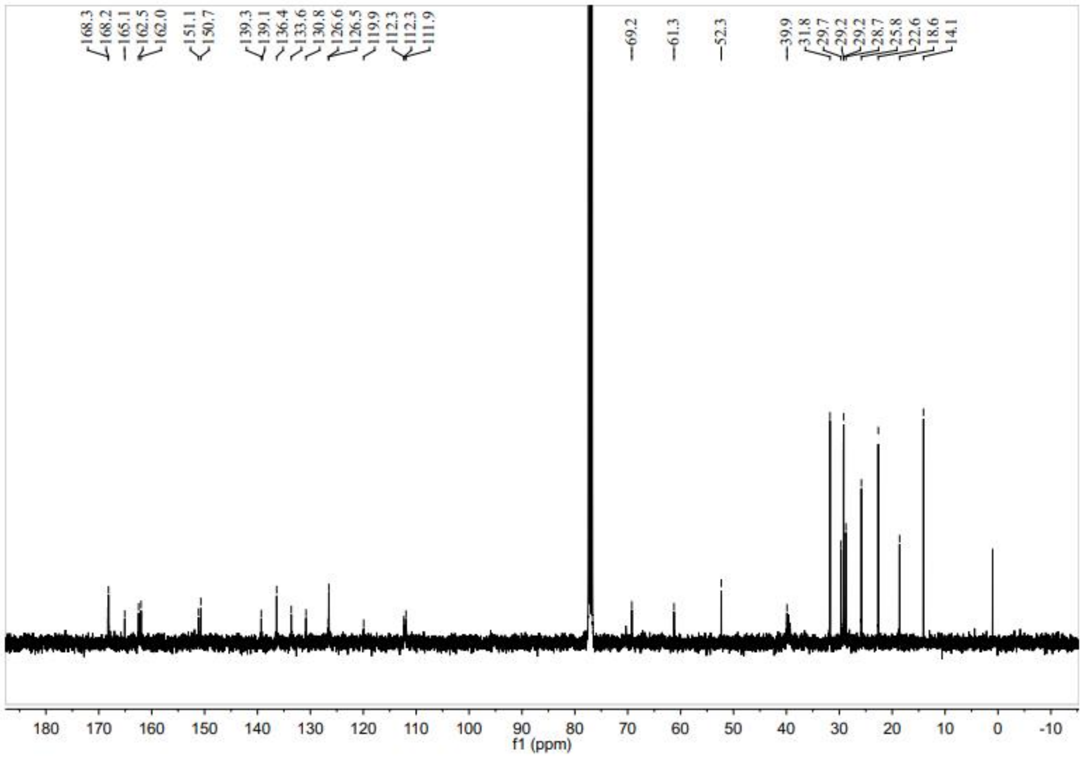

$^{1}H$ NMR (400 MHz, DMSO-$d_6$) spectrum of the macrocycle (**13**).

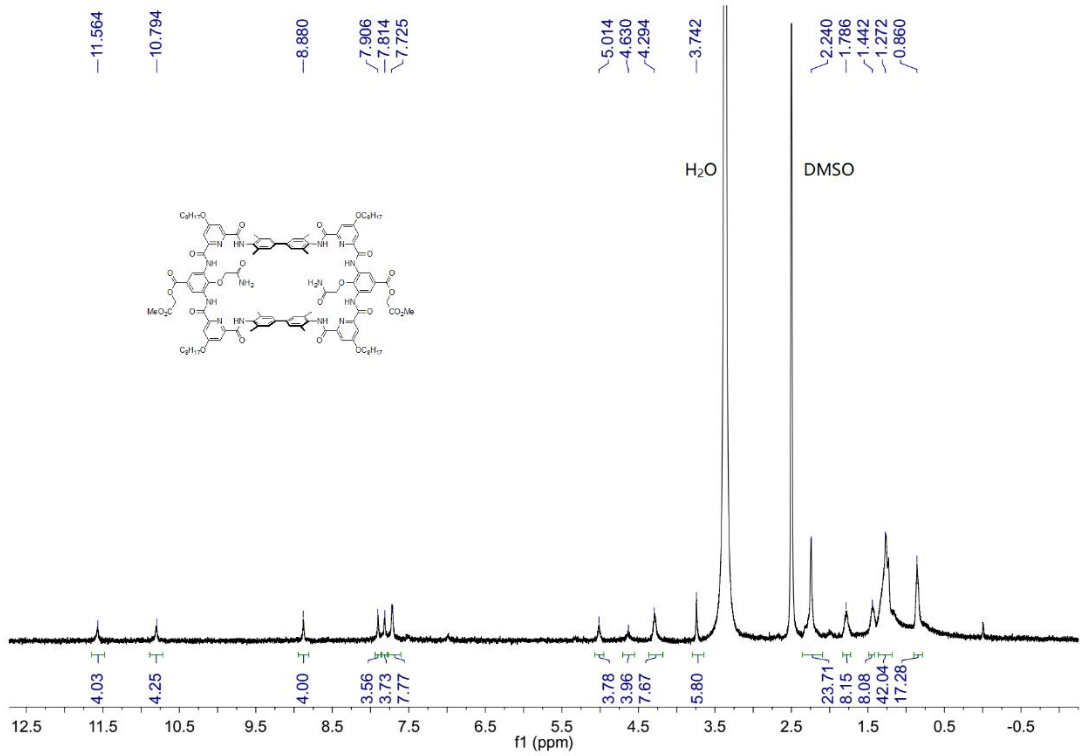


$^{13}C\{^{1}H\}$ NMR (101 MHz, 5%DMSO-$d_6$/$CDCl_3$) spectrum of the macrocycle (**13**).

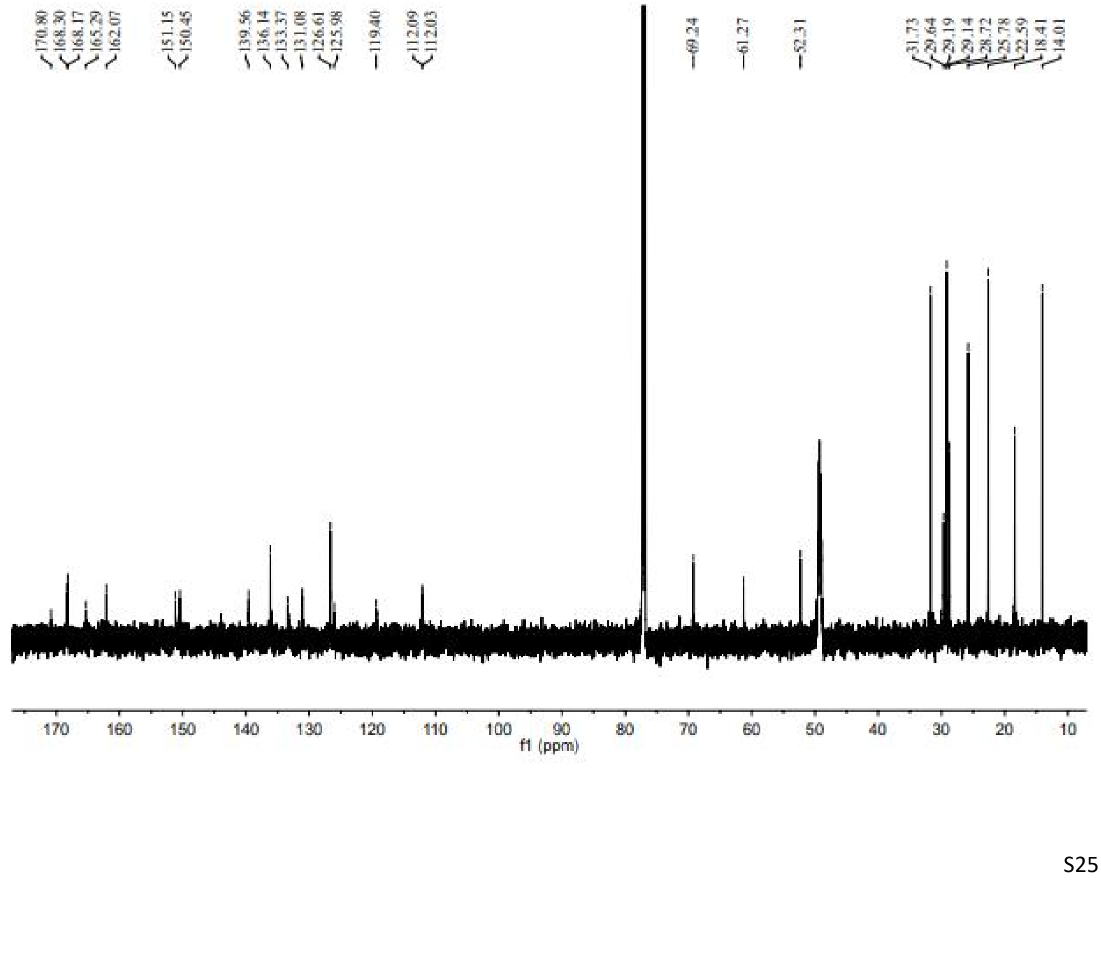

## Mass spectra

HRMS (ESI-TOF) of 2-methoxy-2-oxoethyl 4-hydroxy-3,5-dinitrobenzoate (**2**).

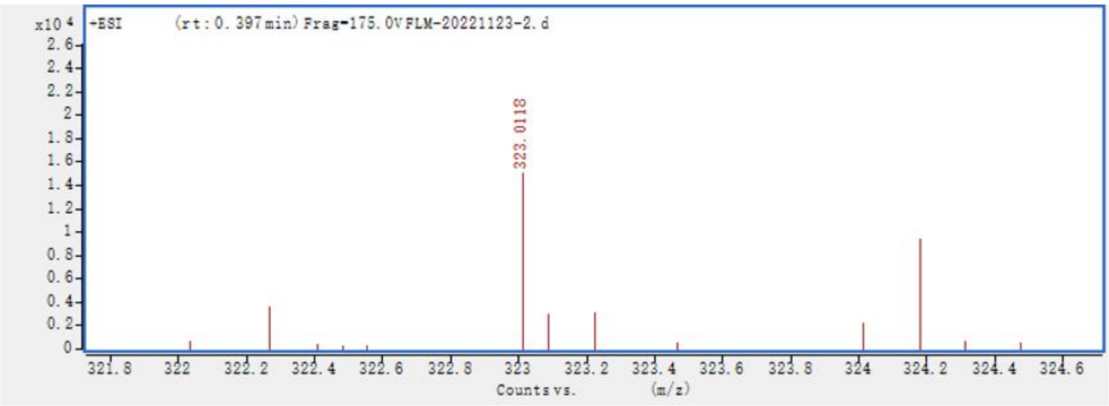


HRMS (ESI-TOF) of the diester (**7**).

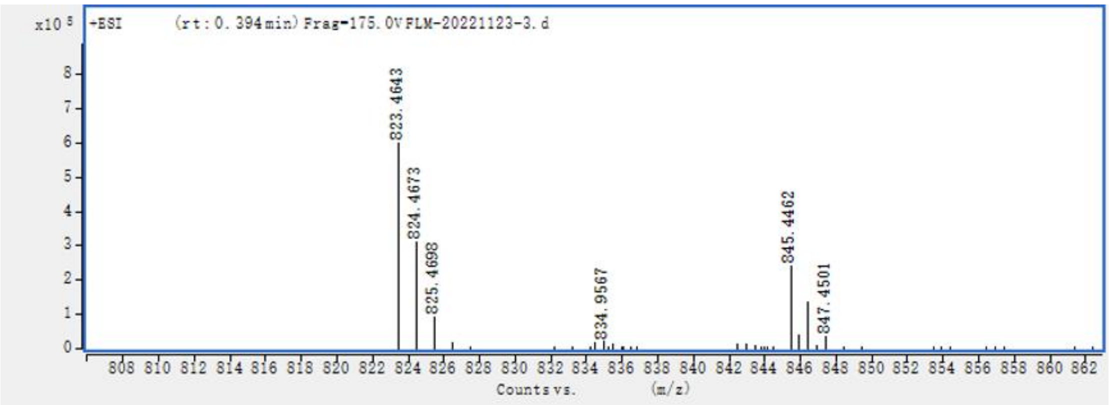


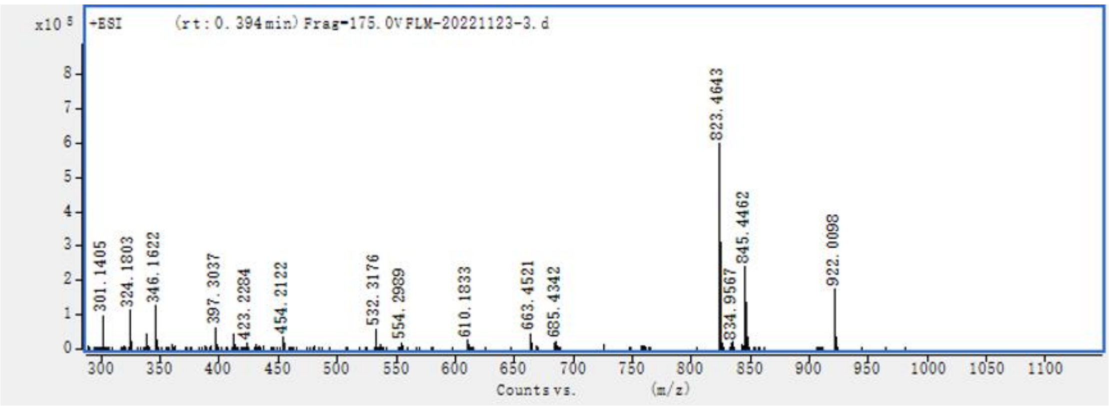

HRMS (ESI-TOF) of the diacid (**8**).

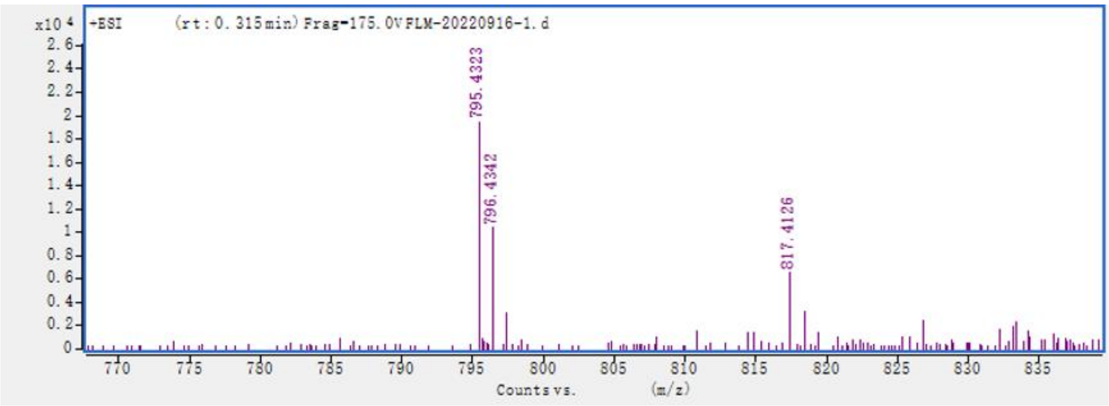


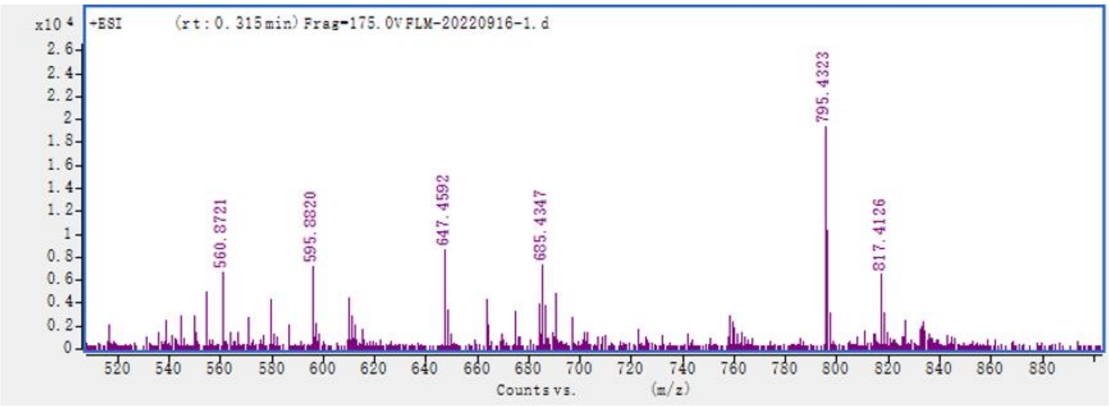

HRMS (ESI-TOF) of the macrocycle (**9**).

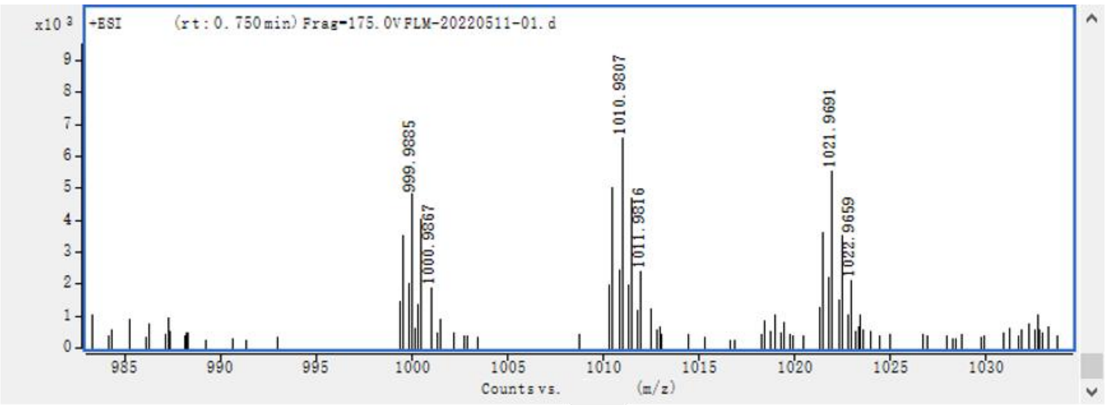


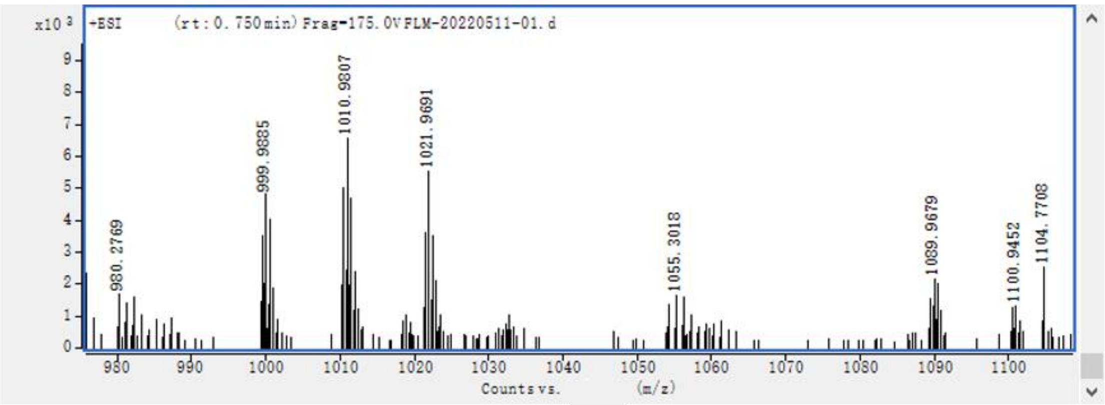

HRMS (ESI-TOF) of the macrocycle (**10**).

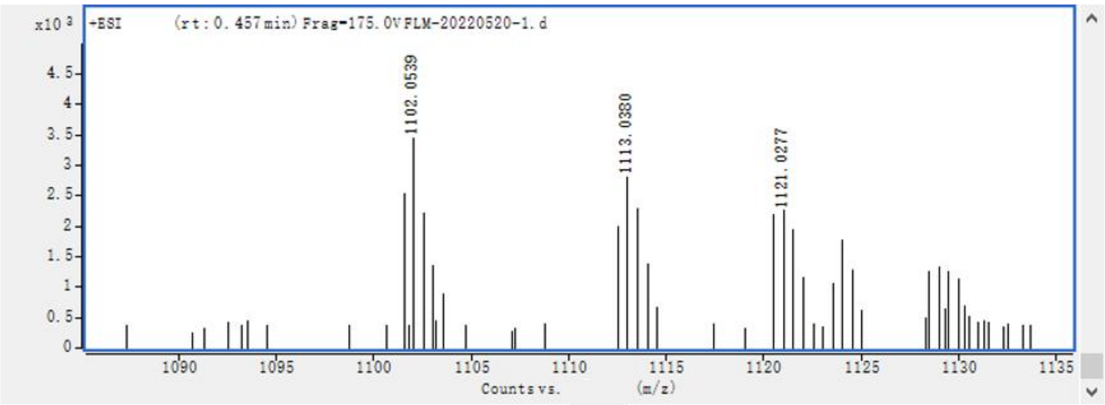


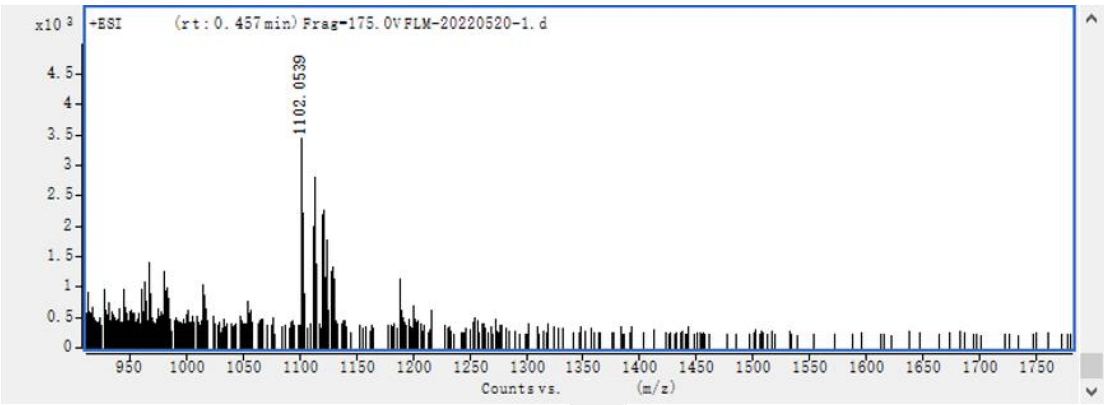

HRMS (ESI-TOF) of the macrocycle (**11**).

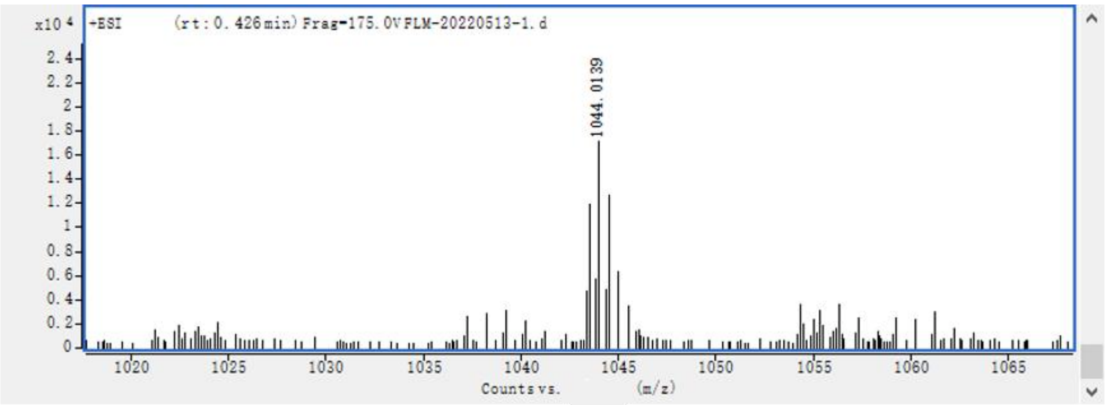


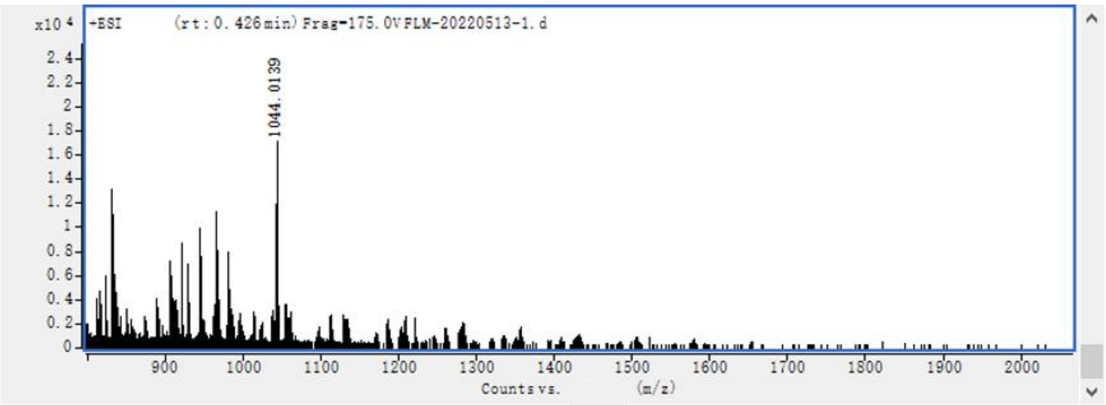

HRMS (ESI-TOF) of the macrocycle (**12**).

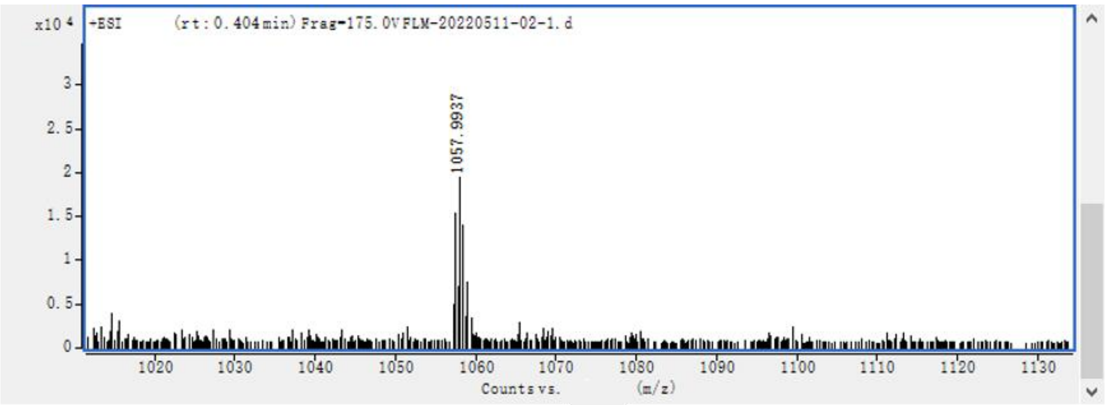


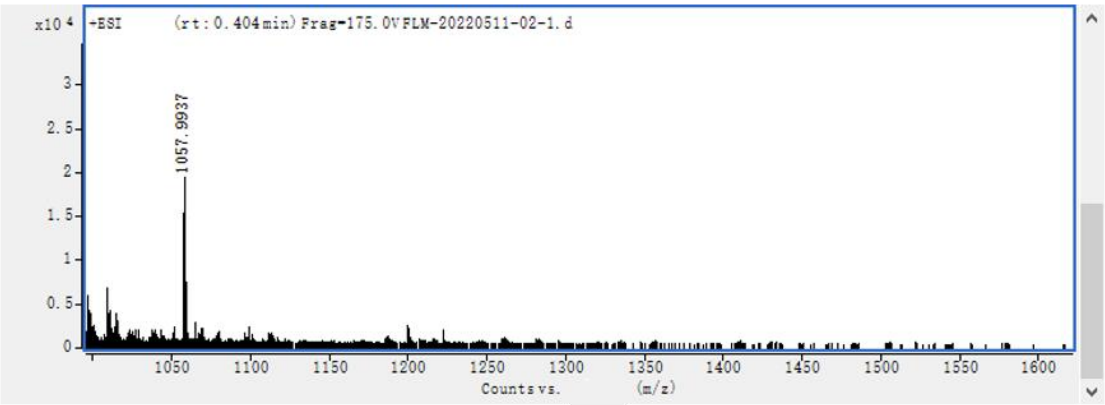

HRMS (ESI-TOF) of the macrocycle (**13**).

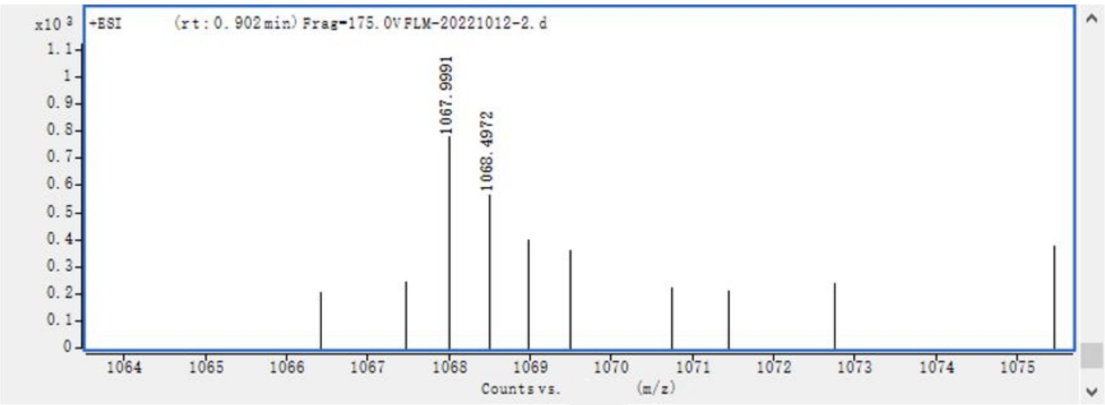


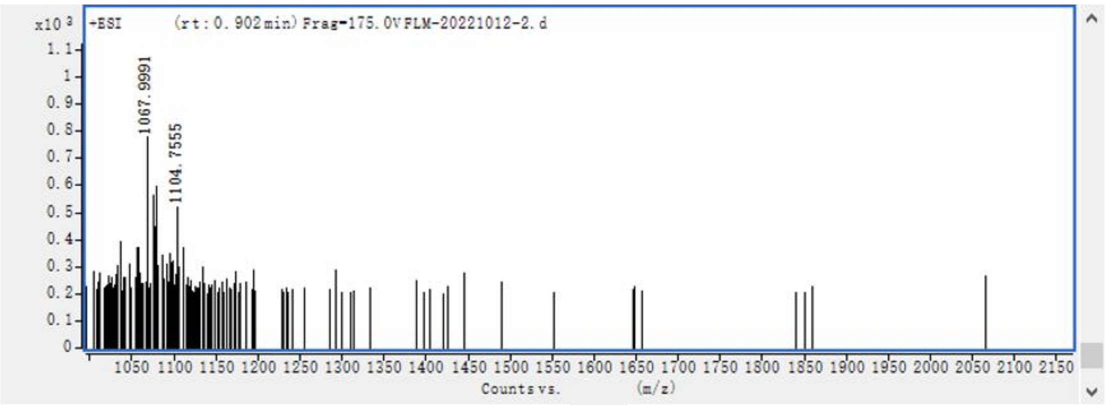

Supplement: Supplementary file 1 [file molecules-28-01338-s001.zip › molecules-2186833-supplementary.pdf]
